# Hybrid manta ray foraging and sine cosine algorithm for managing power transmission congestion influenced by wind energy

**DOI:** 10.1038/s41598-025-13988-z

**Published:** 2025-09-05

**Authors:** Susovan Dutta, Bishaljit Paul, Barnali Kundu, Chandan Kumar Chanda, Kaushik Paul, Pampa Sinha, Hassan Abdurrahman Shuaibu, Taha Selim Ustun

**Affiliations:** 1 Department of Electrical Engineering, Guru Nanak Institute of Technology Kolkata, West Bengal Kolkata, India; 2 Department of Electrical Engineering, Narula Institute of Technology Kolkata, West Bengal Kolkata, India; 3 Department of Electrical Engineering IIEST, Kolkata, West Bengal India; 4 Department of Electrical Engineering, BIT Sindri, Sindri, India; 5 School of Electrical Engineering, KIIT University, Bhubaneswar, 751024 India; 6 Department of Electrical, Telecommunications and Computer Engineering, Kampala International University, Kampala, Uganda; 7 Fukushima Renewable Energy Institute, AIST, Japan Koriyama,

**Keywords:** Power flow control, Renewable energy, Heuristic techniques, Cost minimization, Power rescheduling, Control, Optimization, Electrical and electronic engineering, Energy infrastructure, Renewable energy

## Abstract

This research work proposes a hybrid Manta ray Forging Optimization- Sine Cosine Algorithm (MRFO-SCA) for Congestion Management (CM) that addresses the power system transmission line congestion cost challenges with the integration of Wind Energy System (WES). The proposed method focuses on two key objectives: first, identifying the most influential bus within the power system using the Bus Sensitivity Factor (BSF) to optimally place a wind power source, thereby impacting the power flow in overloaded lines. Second, MRFO-SCA has been developed for optimal power rescheduling of the generators to alleviate congestion while minimizing the congestion cost. The hybrid MRFO-SCA has been formulated by integrating SCA into the MRFO that enhances the exploration and exploitation phases in MRFO leading to the rapid discovery of the global optima. MRFO-SCA has been verified on benchmark functions that have delivered appreciable results. The effectiveness of the proposed approach has been assessed and validated using the IEEE-30 bus system. Simulation results indicate that incorporating WES with MRFO-SCA has led to a reduction in congestion costs by 18.45%, 15.68%, 10.34%, 9.72%, 5.46%, and 1.57% as compared to several recent optimization techniques. A comparative evaluation demonstrates that MRFO-SCA outperforms other methods in terms of congestion cost reduction, system loss minimization, bus voltage improvement, faster convergence, and reduced computational time, making it a more efficient and accurate solution for CM.

## Introduction

The transmission of electrical power is crucial for meeting the energy demands of a power system. Efficient performance of the power system depends on the reliable power transmission that is governed by the system constraints, such as thermal, voltage, and stability limits, effectively. However, there has been significant competition among market participants that may lead to the overburdening of the transmission corridors due to excess power flow beyond their designed facilities. This overloading of the transmission channels may lead to the violations in the system constraints, that may be designated as the congestion in the transmission lines. This scenario of congestion can highly influence the operating state of the power system and its stability. In such situations, the System Operators (SOs) implement regulatory measures in the electricity market and ensure reliable power transactions without exceeding system limits. The continuous increment in the power demand by the consumers and the limited expansion of transmission networks, has issued significant threat towards the congestion in the transmission lines^1^.

The scenario of congestion can arise from various factors, such as excess demand in power from the consumer side, sudden tripping of the generators, unforeseen power flows, and transmission line trips etc. The state of congestion can severely disturb the system reliability, instigate sharp increase in the electricity prices for power transactions, and can affect the continuity in the power transmission to the consumers. It is the system operator’s key responsibility to monitor and address congestion to prevent these issues. Thus, the implementation of effective Congestion Management (CM) strategies is essential for reliable operations. Traditional CM methods that are adopted by the system operators are generator rescheduling, integrating reactive power support, managing consumer demand, implementing demand response programs, and building new transmission infrastructure. The challenge for power system researchers lies in applying these CM techniques at the lowest operational cost, offering considerable potential for improvement through the use of advanced mathematical methods.

## Literature survey

The researchers in the field of power systems have made notable advancements in CM by implementing diverse strategies to prevent and mitigate issues caused by the demand for electricity transmission exceeding network capacity^2,3^. Wang and colleagues explored CM through system frequency monitoring and the integration of distributed energy resources, which also serve as providers of ancillary services^4^. Attar et al. explored CM by proposing a platform to unlock flexibility from various resources through market integration of TSO-DSO coordination, and facilitate data access via a metadata register for improved SO decision-making^5^. Zakaryaseraji and Ghasemi-Marzbali combined the influence distribution generation and the effect of demand response to investigate CM approaches based on the consumer^6^. Roustaei et al. proposed a voltage stability-based CM strategy aimed at improving power flow control while ensuring voltage security within the transmission network^7^. Mishra et al. applied artificial intelligence techniques, including neural networks to address congestion^8^. Dehnavi et al. introduced a novel CM framework based on adapting a segregation and zonal approach in the topology of power system network with restructured electricity market^9^.

In the power system operations, the Generator Rescheduling (GR) serves as a vital strategy for CM by optimizing power generation schedules to mitigate congestion in transmission networks. This process enables better utilization of the already existing infrastructure of the transmission lines, while minimizing the urge for rapid transmission system expansion. By adjusting the output of generators, system operators can relieve congestion, thereby promoting stable operating conditions. Additionally, the combination of the GR and the renewable energy system also provide cost-effective generation dispatch. Subramaniyan and Gomathi used of soft computing methods, such as fuzzy logic and genetic algorithms, to enhance congestion management in transmission networks through GR^10^. Their study highlighted the potential of these techniques to streamline the CM process and achieve efficient power system operation. Similarly, Agrawal et al. proposed a cascaded deep neural network-based framework to facilitate customer participation in CM. Their approach emphasized the role of renewable energy and distributed energy resources (DERs) in optimizing GR, which contributes to congestion relief in deregulated power markets^11^. Chakravarthi et al. included the design of controllers to support efficient generator rescheduling, ensuring the timely mitigation of congestion in power systems^12^. Thiruvel et al. managed congestion that incorporates demand response and accounts for the uncertainties of renewable energy sources. Their strategy also incorporated DG and generator rescheduling to enhance congestion management^13^. Similarly, Saravanan and Anbalagan introduced an intelligent hybrid technique that combined genetic algorithms (GA) with PSO to achieve optimal GR, thereby mitigating congestion in deregulated power markets. Haq et al. introduced a game-theoretic method for CM, leveraging plug-in electric vehicles (PEVs) in conjunction with GR. Their approach involved adjusting the charging and discharging schedules of PEVs to support congestion relief^14^. Verma and Mukherjee developed a CM strategy based on real power GR using an ant lion optimizer. Their approach focused on optimizing generator schedules to alleviate congestion and improve power system performance.

The influence of renewable energy on the power system network plays a significant role in managing the power flow in the transmission line. In^15^, Mouassa et al. introduced a Dwarf Mongoose Optimization Algorithm to address the stochastic optimal power flow in power systems with renewable energy sources, achieving superior performance in minimizing generation costs, transmission losses, and environmental. In another research, Mohamed et al. considered Chaotic African Vultures Optimization Algorithm to maintain optimal power flow incorporating Weibull-based wind power predictions, penalty and reserve costs, and FACTS devices, achieving superior performance over AVOA in cost reduction, power loss minimization^16^. Swirydowicz et al. proposed a GPU-native sparse direct solver for addressing the issue of power flow congestion and economic dispatch considering GPU hardware from both AMD and NVIDIA to accelerate sparse linear system computations while achieving significant performance improvements and highlighting opportunities for further optimization in heterogeneous computing environments^17^. Gracia et al. studied the architecture of the bipolar DC grid to control the power flow and alleviate congestion with asymmetric loading, transforming a nonlinear programming (NLP) model^18^. Naderi et al. proposed a hybrid wavelet mutation-based algorithm to solve the congestion with multi-fuel constraints, optimizing generation cost, emissions, power loss, and voltage deviation^19^. Khani et al. proposed a bi-level stochastic model for integrated energy management in interconnected transmission and distribution networks, incorporating renewable energy to reduce the risk of overloading in the transmission network^20^. Ullah et al. proposed a hybrid PSO-GSA algorithm for optimal energy trading in interconnected microgrids (MGs) for optimizing the power flow considering the influence uncertainties in the renewable energy utilization^21^.

The operation of competitive energy markets has driven the need for advanced optimization algorithms that effectively address power system challenges, yielding accurate and improved results. Traditionally, power system researchers have relied on deterministic methods, including classical approaches, to solve the optimal power generation and dispatch problem. These issues have been tackled using various traditional techniques in convex optimization. However, the use of these deterministic methods is limited by constraints. In such cases, the solutions derived from these methods are highly dependent on the initial conditions, which may lead to local optima. To overcome these limitations, stochastic methods offer a more efficient solution by bypassing the issues inherent in deterministic approaches. This has led to the adoption of heuristic and metaheuristic techniques, which are better equipped to provide optimal solutions compared to deterministic methods. Many researchers have turned to these techniques to find effective solutions for optimal power generation, ensuring a reliable and sustainable power flow through transmission networks^22^. The application of the heuristics techniques for the optimal operation of the power system can be found in the areas like system risk mitigation in competitive systems with artificial gorilla troops^23^, IPD-(1 + I) controller for frequency control in a power system with sine-cosine algorithmic technique^24^, particle swarm optimization for maximum power point tracking in inverters^25^. A well-chosen selection and proper tuning of control parameters in meta-heuristic methods significantly influence the quality of the solutions obtained. These techniques offer a practical approach to finding optimal solutions while reducing computational effort. The primary objective of this study is to design and implement an effective optimization strategy aimed at addressing the CM problem, with a focus on achieving substantial cost reduction in the final outcome.

In this research, the Manta Ray Forge Optimization (MRFO) has been introduced with the hybridization of Sine Cosine Algorithm (SCA) for obtaining better output by avoiding the MRFO getting trapped in the local optima. The selection of MRFO and SCA for this research has been based on the “No Free Lunch” (NFL) theorems for optimization assert that no optimization algorithm can outperform all others across every possible problem^26^. In essence, if an algorithm shows strong performance on a particular group of problems, it must, on average, perform less effectively on other problems. According to this Manta-Ray Forge Optimization (MRFO) has contributed appreciable outcome for the power system optimization problem like ELD^27^, optimal sizing of renewable energy^28^ Load frequency control^29^, DG placement^30^, power transfer capabilities^31^but in many cases it has struggled to in its exploration and exploitation phases and have confined the results in the region of local optima. Moreover, in addition to this, it has been observed that the Sine Cosine Algorithm (SCA) has also delivered significant improved results in the field of power system like LFC^32^, ELD^33^, Parameter estimation solar PV^34^ reactive power dispatch^35^ and it has been also used a part of hybridization to improve the phases of exploration and exploitation in several recent optimization techniques that has delivered appreciable outcome like in the field of its SCA-Jaya optimization for OPF^36^, SCA-Chimp optimization based feature selection^37^, automatic generation control system based on hybrid Aquila Optimizer-SCA^38^, reactive power optimization^39^. From these literatures it can be considered that the inclusion of the features of SCA in the MRFO to form a hybrid MRFO-SCA that can also deliver significant improvement in the results for the considered CM problem with the application of the proposed MRFO-SCA.

This study introduces a hybrid Manta Ray Forge Optimization-Sine Cosine Algorithm (MRFO-SCA) to address the CM cost optimization challenge, aiming to efficiently manage power generation while integrating a WES to reduce congestion costs. The optimal location for WES integration is identified using Bus Sensitivity Factors (BSF). Incorporating WES not only mitigates power flow violations but also reduces real power losses and enhances system voltage.

The MRFO-SCA has been applied to the IEEE 30-bus system to evaluate its effectiveness in minimizing CM costs while reducing system losses and improving voltage levels. Furthermore, a statistical comparison of MRFO-SCA with other optimization techniques has been conducted to demonstrate its efficiency. For additional benchmarking, the CM problem has been solved using Firefly Algorithm (FFA), Particle Swarm Optimization (PSO), African Vulture Optimization Algorithm (AVOA), Sine-Cosine Algorithm(SCA), Aquila Optimizer (AO), and MRFO, showcasing the relative performance of the MRFO-SCA.

### Contributions

- A congestion management (CM) strategy has been formulated by considering the impact of the Wind Energy System (WES). Bus Sensitivity Factors (BSF) are employed to determine the most suitable location for integrating the WES.
- Hybrid Manta Ray Forge Optimization-Sine Cosine Algorithm (MRFO-SCA) has been proposed for addressing the CM problem, incorporating the features of SCA in the MRFO to enhance both the exploration and exploitation phases, resulting in an improved convergence rate.
- MRFO-SCA is further extended as a robust optimization technique to minimize costs in the CM problem, focusing on optimal power management under the influence of the WES.
- The effectiveness and validation of the proposed CM approach are demonstrated using the IEEE-30 bus system. Additionally, the CM problem is solved using FFA, PSO, AVOA, AO, SCA, and MRFO to provide a comparative performance analysis with MRFO-SCA. The comparison evaluates MRFO-SCA in terms of congestion cost minimization, convergence behavior, voltage profile improvement, system loss reduction, and computational efficiency.

## Problem formulation

### Bus sensiitvity factor

The Bus Sensitivity Factor (BSF) has been considered in this research to identify the optimal placement of Wind Energy System (WES) for power injection to alleviate congestion in transmission lines. The BSF represents the change in power flow ($\Delta {P_{ij}}$) through a congested line *k* located between buses *i* and *j*, caused by an injection of power ($\Delta {P_n}$) at bus *n*. Mathematically, this relationship is expressed in Eq. (1)

1 $$BSF_{k}^{n}=\frac{{\Delta {P_{ij}}}}{{\Delta {P_n}}}$$

The computation of the BSF has been illustrated as follows:

2 $${P_{ij}}= - V_{i}^{2}{Y_{ij}}\cos {\theta _{ij}}+{V_i}{V_j}{Y_{ij}}\cos ({\theta _{ij}}+{\delta _j} - {\delta _i})$$

3 $$\Delta {P_{ij}}=\frac{{\partial {P_{ij}}}}{{\partial {\delta _i}}}\Delta {\delta _i}+\frac{{\partial {P_{ij}}}}{{\partial {\delta _j}}}\Delta {\delta _j}+\frac{{\partial {P_{ij}}}}{{\partial {V_i}}}\Delta {V_i}+\frac{{\partial {P_{ij}}}}{{\partial {V_j}}}\Delta {V_j}$$

The Eq. (3) can be represented as:

4 $$\Delta {P_{ij}}={\tau _{ij}}\Delta {\delta _i}+{\rho _{ij}}\Delta {\delta _j}+{\chi _{ij}}\Delta {V_i}+{\varepsilon _{ij}}\Delta {V_j}$$

Here.

5 $${\tau _{ij}}={V_i}{V_j}{Y_{ij}}\sin ({\theta _{ij}}+{\delta _j} - {\delta _i})$$

6 $${\rho _{ij}}= - {V_i}{V_j}{Y_{ij}}\sin ({\theta _{ij}}+{\delta _j} - {\delta _i})$$

7 $${\chi _{ij}}={V_j}{Y_{ij}}\cos ({\theta _{ij}}+{\delta _j} - {\delta _i}) - 2{V_i}{Y_{ij}}\cos {\theta _{ij}}$$

8 $${\varepsilon _{ij}}={V_i}{Y_{ij}}\cos ({\theta _{ij}}+{\delta _j} - {\delta _i})$$

From the load flow studies we know that:

9 $$\left[ {\begin{array}{*{20}{c}} {\Delta P} \\ {\Delta Q} \end{array}} \right]=\left[ J \right]\left[ {\begin{array}{*{20}{c}} {\Delta \delta } \\ {\Delta V} \end{array}} \right]=\left[ {\begin{array}{*{20}{c}} {{J_{11}}}&{{J_{12}}} \\ {{J_{21}}}&{{J_{22}}} \end{array}} \right]\left[ {\begin{array}{*{20}{c}} {\Delta \delta } \\ {\Delta V} \end{array}} \right]{\text{ }}$$

Neglecting the ∆P-∆V and ∆Q-∆δ coupling the Eq. (9) can be represented as in Eqs. (10) and (11):

10 $$\left[ {\Delta P} \right]=\left[ {{J_{11}}} \right]\left[ {\Delta \delta } \right]{\text{ }}$$

11 $$\left[ {\Delta Q} \right]=\left[ {{J_{22}}} \right]\left[ {\Delta V} \right]{\text{ }}$$

From Eq. (10),

12 $$\Delta \delta ={\left[ {{J_{11}}} \right]^{ - 1}}\left[ {\Delta P} \right]=\left[ M \right]\left[ {\Delta P} \right]$$

Equation (3) can be represented as:

13 $$\Delta {P_{ij}} = {\tau _{ij}}{\delta _i} + {\rho _{ij}}{\delta _j}i = 2,n,N(slack{\mkern 1mu} bus{\mkern 1mu} is{\mkern 1mu} taken{\mkern 1mu} as{\mkern 1mu} 1)$$

14 $$\Delta {\delta _i}=\sum\limits_{{l=2}}^{N} {{m_{il}}\Delta {P_l}}$$

Substituting Eq. (13) in (14):

15 $$\Delta {P_{ij}}={\tau _{ij}}\sum\limits_{{l=2}}^{N} {{m_{il}}\Delta {P_l}+{\rho _{ij}}} \sum\limits_{{l=2}}^{N} {{m_{jl}}\Delta {P_l}}$$

16 $$\begin{gathered} \Delta {P_{ij}}=({\tau _{ij}}{m_{i1}}+{\rho _{ij}}{m_{j1}})\Delta {P_1}+({\tau _{ij}}{m_{i2}}+{\rho _{ij}}{m_{j2}})\Delta {P_2} \hfill \\ +....+({\tau _{ij}}{m_{in}}+{\rho _{ij}}{m_{jn}})\Delta {P_n} \hfill \\ \end{gathered}$$

Equation (16) can be represented as:

17 $$\Delta {P_{ij}}=BSF_{1}^{k}\Delta {P_1}+BSF_{2}^{k}\Delta {P_2}+....+BSF_{n}^{k}\Delta {P_n}$$

Thus, the BSF can be portrayed as:

18 $$BSF_{n}^{k}=({\tau _{ij}}{m_{in}}+{\rho _{ij}}{m_{jn}})$$

The Bus Sensitivity Factors (BSFs) for the buses can be calculated using Eq. (28). Placing the WES at a load bus can facilitate CM. By analyzing the BSF values, the most sensitive buses, which are capable of alleviating transmission line congestion through adjustments in power injection, can be identified. Buses exhibiting the most significant deviations in BSF values are considered highly sensitive to changes in power flow, making them ideal candidates for the installation of the WES.

The BSF signifies the sensitivity at a particular bus that states the ratio of the change in the power flow in the congested line with respect to the small power injection at a certain bus. This can be understood as if a small amount of power is injected by the renewable energy sources at a particular bus within the system, then how that small amount of injected power will affect the change in the power flow in the congested line. The representation of the mathematical model of BSF has been given in Eq. (1). The BSF values are computed for all the load buses with respect to the congested line power flow and the values are presented in Table 4 and it is seen that Bus 20 has the most deviated BSF values for the congested lines. So, from the mathematical expression of BSF it can be seen that the bus that has the most deviated values of the BSF will have a greater impact on the power flow on the congested line when the power from the renewable energy is injected at that particular bus (that has the highest deviated/higher value of BSF). Thus, the BSF acts as an indicator to choose the bus for the placement of the wind farm to control the power flow in the congested lines.

### Wind energy system modelling

This research examines the variability of wind speed utilizing the Weibull Probability Density Function (PDF) as described in^40^. In this context, wind speed modeling is conducted using the Weibull PDF, expressed as follows:

19 $${f_v}(v) = \left( {\frac{k}{c}} \right) * {\left( {\frac{v}{c}} \right)^{(k - 1)}}\exp \left[ { - {{\left( {\frac{v}{c}} \right)}^k}} \right]$$

In Eq. (19), the parameters *c* and *k* are referred to as the scale factor and shape factor, respectively, both of which are positive values. WES power generation can be divided into three distinct scenarios. The first scenario occurs when the wind speed is below the cut-in speed, during which the WES produces no power output. The second scenario takes place when the wind speed falls between the cut-in speed and the rated speed, resulting in power output increasing linearly. This phase is often referred to as the continuous zone. Lastly, when the wind speed exceeds the rated speed but remains below the cutout speed, the WES delivers power at its rated capacity. This power generation process is characterized by a discrete probability distribution function. Similarly, no power is generated when the wind speed is either below the cut-in speed or above the cutout speed. The power output of the WES can be expressed as follows:

20 $$p = \left\{ {\begin{array}{*{20}{c}} {p = 0}\\ {p = {p_r}}\\ {p = {p_r}} \end{array} * \frac{{(v - {v_1})}}{{({v_1} - {v_1})}}\begin{array}{*{20}{c}} {for{v_1} < vandv> {v_0}}\\ {for{v_1} \le v \le {v_r}}\\ {for{v_1} \le v \le {v_0}} \end{array}} \right\}$$

The Weibull PDF is represented as:

21 $${f_v}(p) = \left( {\frac{{k({v_r} - {v_r})}}{{{c^k} * {p_r}}}} \right) * \left[ {{v_i} + \frac{p}{{{p_i}}}{{({v_r} - {v_i})}^{k - 1}}} \right]\exp \left[ { - {{\left( {\frac{{{v_i} + \frac{p}{{{p_i}}}({v_r} - {v_i})}}{c}} \right)}^k}} \right]$$

The Weibull probability density function (PDF) is used for wind speed estimation because it effectively models the statistical distribution of wind speeds over time essential for energy forecasting. The wind speed in any geographic region is intermittent in nature. Due to this the wind speeds in most locations do not follow a normal distribution. Weibull distribution closely matches actual wind speed frequency patterns observed in nature. The Wind power output depends on the cube of wind speed. Weibull distribution allows accurate estimation of average power output from a wind turbine over time.

The velocity of the wind in a specific region is intermittent. The velocity is generated considering the Weibull distribution implementation to model how frequently different wind speeds occur. This is then combined with the turbine’s power curve to predict energy output. The Weibull PDF and the wind speed data have been adapted in this research based on the reference^41^.

### Congestion cost formulation

The process of rescheduling power aims to minimize congestion costs by prioritizing the bids provided by system generators. Consequently, a fundamental objective of the proposed congestion management approach is to achieve the lowest possible rescheduling cost, which can be represented mathematically as follows:

22 $$RC=\sum\limits_{{i=1}}^{{{N_g}}} {(R_{i}^{u}\Delta P_{{gi}}^{u}+} R_{i}^{d}\Delta P_{{gi}}^{d})+{R_w}{P_w}$$

In Eq. (22) $\Delta P_{{gi}}^{u}$ and $\Delta P_{{gi}}^{d}$denotes the incremental and decremental rescheduled power. The real power output from the WES is denoted as ${P_w}$, with a maximum capacity of 45 MW with wind speed 10 m/sec. The terms$R_{i}^{u}$and$R_{i}^{d}$represent the incremental and decremental bids submitted by the power generation units, respectively, while$R_{w}^{{}}$indicates the price bid associated with the WES. The WES output for a specific level of wind speed is calculated using Eq. (20)

*Inequality Constraints*:

Real power generation constraints:

23 $$\Delta P_{{gi}}^{{\hbox{min} }} \leqslant \Delta {P_{gi}} \leqslant \Delta P_{{gi}}^{{\hbox{max} }}$$

24 $${P_{gi}} - P_{{gi}}^{{\hbox{min} }}=\Delta P_{g}^{{\hbox{min} }}\& P_{{gi}}^{{\hbox{max} }} - {P_{gi}}=\Delta P_{{gi}}^{{\hbox{max} }}$$

Voltage limit constraints:

25 $$V_{i}^{{\hbox{min} }} \leqslant {V_i} \leqslant V_{i}^{{\hbox{max} }}$$

Reactive power generation constraints:

26 $$Q_{{gi}}^{{\hbox{min} }} \leqslant {Q_{gi}} \leqslant Q_{{gi}}^{{\hbox{max} }}$$

Power flow limits for transmission lines:

27 $${F_t} \leqslant F_{t}^{{\hbox{max} }}$$

Equality Constraints:

28 $${P_i}={V_i}\sum\limits_{{i=1}}^{N} {{V_j}[{G_{ij}}\cos ({\delta _i} - {\delta _j})+{B_{ij}}\sin ({\delta _i} - {\delta _j})}$$

29 $${Q_i}={V_i}\sum\limits_{{i=1}}^{N} {{V_j}[{G_{ij}}\sin ({\delta _i} - {\delta _j})+{B_{ij}}\sin ({\delta _i} - {\delta _j})}$$

Equations (23) and (24) represent the system’s power balance equations. Here, *N* indicates buses in the network. The active and reactive power injections at the *i*^*th*^ bus are denoted by *P*_*i*_ and *Q*_*i*_ respectively. *V*_*i*_ and *V*_*j*_ correspond to the voltage magnitudes, while *δ*_*i*_ and *δ*_*j*_ represent the phase angles at buses i and j, respectively. The conductance and susceptance between buses *i* and *j* are denoted by *Gij* and *Bij* respectively. Equation (25) defines the voltage limits for the buses. Equation (27) specifies the line power flow constraints, with $F_{t}^{{\hbox{max} }}$and ${F_t}$​indicating the maximum allowable power flow and the current power flow in the system, respectively.

## Formulation of hybrid manta ray forge optimization-sine cosine algorithm

### Sine cosine algorithm

The Sine Cosine Algorithm (SCA), introduced by Mirjalili, utilizes a mathematical framework grounded in sine and cosine functions to create a diverse set of initial random solutions. This approach allows these solutions, referred to as agents, to move either away from or closer to the optimal solution. The exploration and exploitation processes within the algorithm can be expressed mathematically as follows:

30 $$Z_{i}^{{t+1}}=Z_{i}^{t}+{\lambda _{rand\_1}} \times \sin ({\lambda _{rand\_}}_{2}) \times \left| {{\lambda _{rand\_3}}.Pos_{i}^{t} - Z_{i}^{t}} \right|$$

31 $$Z_{i}^{{t+1}}=Z_{i}^{t}+{\lambda _{rand\_1}} \times \cos ({\lambda _{rand\_}}_{2}) \times \left| {{\lambda _{rand\_3}}.Pos_{i}^{t} - Z_{i}^{t}} \right|$$

In Eqs. (30) and (31), the term represents the current position of the solution in the i^th^ dimension, while *t* denotes the iteration number. The variables $Ran{d_1}$,$Ran{d_2}$, and $Ran{d_3}$ ∈ [0,1 are random values generated]. During the exploration and exploitation phases, the condition $0.5 \leqslant Ran{d_4}<0.5$ is applied. These two equations can be unified and expressed as a single representation.

32 $$Z_{i}^{{t+1}}=\left\{ {\begin{array}{*{20}{c}} {Z_{i}^{t}+{\lambda _{rand\_}}_{1} \times \sin ({\lambda _{rand\_}}_{2}) \times \left| {{\lambda _{rand\_}}_{3}.Pos_{i}^{t} - Z_{i}^{t}} \right|,{\lambda _{rand\_}}_{4}<0.5} \\ {Z_{i}^{t}+{\lambda _{rand\_}}_{1} \times \cos ({\lambda _{rand\_}}_{2}) \times \left| {{\lambda _{rand\_}}_{3}.Pos_{i}^{t} - Z_{i}^{t}} \right|,{\lambda _{rand\_4}} \geqslant 0.5} \end{array}} \right.$$

In the Sine Cosine Algorithm (SCA), the balance between the exploration and exploitation phases is maintained by adjusting the sine and cosine ranges, as outlined in Equations (30) to (32). This adjustment is guided by the following equation:

33 $${\lambda _{rand\_}}_{1}={B_c} - {t_c}\frac{B}{{{t_{\hbox{max} }}}}$$

Here in Eq. (33), ${t_{\hbox{max} }}$,${t_c}$, and ${B_c}$are maximum iterations, current iterations and a constant value respectively.

### Manta ray foraging optimization (MRFO)

MRFO algorithm has been introduced by Zhao et al. in 2020. The algorithm has been inspired by the foraging behavior of manta rays. The algorithm’s structure mimics three specific foraging strategies exhibited by manta rays: chain foraging, cyclone foraging, and somersault foraging. MRFO initializes its population randomly within the boundaries of the search space. The population is then updated using the three foraging strategies, which guide the optimization process.

#### Chain foraging

Manta rays form a structured line by linking their heads and tails, creating a foraging chain. In the context of the MRFO algorithm, the optimal solution represents areas with a higher density of plankton, symbolizing the global solution. In the initial stage of the foraging chain, the leading manta ray traces the path towards the planktons and is followed by the group manta rays. This behavior, referred to as chain foraging, can be expressed as follows:

34 $$x_{i}^{{t+1}}=\left\{ {\begin{array}{*{20}{c}} \begin{gathered} x_{i}^{t}+{r_1}(x_{{best}}^{t} - x_{i}^{t})+ \hfill \\ \alpha (x_{{best}}^{t} - x_{i}^{t}),{\text{ }}i=1{\text{ }} \hfill \\ \end{gathered} \\ \begin{gathered} x_{i}^{t}+{r_2}(x_{{i - 1}}^{t} - x_{i}^{t})+ \hfill \\ \alpha (x_{{best}}^{t} - x_{i}^{t}),{\text{ }}i=2,3,....NP \hfill \\ \end{gathered} \end{array}} \right.$$

35 $$\alpha =2 \cdot {r_3} \cdot \sqrt {\left| {\log ({r_4})} \right|}$$

In Eq. (34), represents the position of an individual at a specific generation. The term refers to a random vector with values in the range [0,1], where =1,2,3,4. The $x_{{best}}^{t}$variable indicates the best solution. $NP$resembles the population size, and$\alpha$is weighted coefficient.

##### Cyclone foraging

When manta rays detect plankton beneath the ocean surface, they traverse in the direction of their food source in a spiral formation, maintaining a coordinated chain by following the movements of the individual in front. The mathematical model representing this cyclone foraging behavior is as follows:

36  $$x_{i}^{{t+1}}=\left\{ {\begin{array}{*{20}{c}} \begin{gathered} x_{{best}}^{t}+{r_5}(x_{{best}}^{t} - x_{i}^{t})+ \hfill \\ \beta (x_{{best}}^{t} - x_{i}^{t}),{\text{ }}i=1{\text{ }} \hfill \\ \end{gathered} \\ \begin{gathered} x_{{best}}^{t}+{r_6}(x_{{i - 1}}^{t} - x_{i}^{t})+ \hfill \\ \beta (x_{{best}}^{t} - x_{i}^{t}),{\text{ }}i=2,3,....NP \hfill \\ \end{gathered} \end{array}} \right.$$

37 $$\beta =2 \cdot \exp ({r_7} \cdot (ite{r_{\hbox{max} }} - iter+1)/ite{r_{\hbox{max} }}) \cdot \sin (2\pi {r_7})$$

In Eq. (36) r is random value ∈ [0,1], and i = 5,6 denotes specific indices. The term $\beta$ indicates the weighting coefficient.

Equation (36) demonstrates that the food source serves as the central point for spiral foraging, enabling effective exploitation of the search space near the food. To enhance the search capacity, a randomly generated position within the defined search region is selected as the new center for spiral foraging. This helps manta rays explore better areas beyond their current optimal position.

In the MRFO framework, the random spiral foraging mechanism focuses primarily on exploration, fostering a global search capability. The mathematical representation of this concept is as follows:

38 $${x_{rand}}=lb+{r_8} \cdot (ub - lb)$$

39 $$x_{i}^{{t+1}}=\left\{ {\begin{array}{*{20}{c}} \begin{gathered} x_{{rand}}^{{}}+{r_9}(x_{{best}}^{t} - x_{i}^{t})+ \hfill \\ \beta (x_{{best}}^{t} - x_{i}^{t}),{\text{ }}i=1{\text{ }} \hfill \\ \end{gathered} \\ \begin{gathered} x_{{rand}}^{{}}+{r_{10}}(x_{{i - 1}}^{t} - x_{i}^{t})+ \hfill \\ \beta (x_{{best}}^{t} - x_{i}^{t}),{\text{ }}i=2,3,....NP \hfill \\ \end{gathered} \end{array}} \right.$$

In Eq. (39),${x_{rand}}$represents a randomly selected position, while ${r_i} \in [0,1]$defines the search space boundaries. The upper limit of the search area is denoted by $ub$ and the lower limit is represented by $lb$.

##### Somersaulting foraging

At this stage, the food source is considered the central point or pivot. Each manta ray moves in a circular motion around this pivot, exploring new locations. This process is mathematically expressed as:

40 $$x_{i}^{{t+1}}=x_{i}^{t}+S \cdot ({r_{11}} \cdot x_{{best}}^{t} - {r_{12}} \cdot x_{i}^{t}),{\text{ }}i=1,2,....NP$$

In Eq. (40), *S* represents the somersault factor, which determines the range of movement for the manta rays during the somersault process. *S* is assigned a value of 2. The parameters ${r_{11}}$*and* ${r_{rr}}$​ ∈[0,1].

### Hybrid Mantaray Forge algorithm and sine cosine algorithm

The MRFO algorithm faces challenges such as limited exploitation capability, reduced population diversity, and a tendency to become stuck in local optima. These issues primarily arise from an imbalance between the exploration and exploitation phases. To address the limitations of the optimization method and improve its performance, the MRFO and SCA algorithms are combined to form the hybrid MRFO-SCA approach.

In the standard MRFO algorithm, during the early stages of execution the search agents update their positions to align with the latest best position, as defined by Eq. (34). However, since the optimal position of the candidate solution is initially unknown, this update mechanism can result in convergence toward local optima.Furthermore, during the MRFO’s search process, large step changes in the position vector can hinder effective exploration of the search space. To address these challenges, the SCA has been introduced in the MRFO to improve the algorithm’s performance. The updated chain foraging mathematical formulation for the MRFO-SCA is expressed as:

 41 $$x_{{i(\bmod ified)}}^{{t+1}}=\left\{ {\begin{array}{*{20}{c}} \begin{gathered} (x_{i}^{t}+{r_1}\operatorname{Sin} ({\lambda _{rand\_}}_{2}){\lambda _{rand\_}}_{3}(x_{{best}}^{t} - x_{i}^{t})+ \hfill \\ \alpha \operatorname{Sin} ({\lambda _{rand\_}}_{2}){\lambda _{rand\_}}_{3}(x_{{best}}^{t} - x_{i}^{t})),{\text{ }}i=1{\text{ }} \hfill \\ \end{gathered} \\ \begin{gathered} (x_{i}^{t}+{r_2}\operatorname{Sin} ({\lambda _{rand\_}}_{2}){\lambda _{rand\_}}_{3}(x_{{i - 1}}^{t} - x_{i}^{t})+ \hfill \\ \alpha \operatorname{Sin} ({\lambda _{rand\_}}_{2}){\lambda _{rand\_}}_{3}(x_{{best}}^{t} - x_{i}^{t})),{\text{ }}i=2,3,....NP \hfill \\ \end{gathered} \end{array}} \right.$$

42 $$x_{{i(\bmod ified)}}^{{t+1}}=\left\{ {\begin{array}{*{20}{c}} \begin{gathered} (x_{i}^{t}+{r_1}\operatorname{Cos} ({\lambda _{rand\_}}_{2}){\lambda _{rand\_}}_{3}(x_{{best}}^{t} - x_{i}^{t})+ \hfill \\ \alpha \operatorname{Cos} ({\lambda _{rand\_}}_{2}){\lambda _{rand\_}}_{3}(x_{{best}}^{t} - x_{i}^{t})),{\text{ }}i=1{\text{ }} \hfill \\ \end{gathered} \\ \begin{gathered} (x_{i}^{t}+{r_2}\operatorname{Cos} ({\lambda _{rand\_}}_{2}){\lambda _{rand\_}}_{3}(x_{{i - 1}}^{t} - x_{i}^{t})+ \hfill \\ \alpha \operatorname{Cos} ({\lambda _{rand\_}}_{2}){\lambda _{rand\_}}_{3}(x_{{best}}^{t} - x_{i}^{t})),i=2,3,....NP \hfill \\ \end{gathered} \end{array}} \right.$$

The hybridization of SCA with MRFO allows the manta rays to move in smaller steps toward the target location while efficiently exploring within the boundaries of the search area. The enhanced chain foraging mechanism is described by Eqs. (41) and (42).

To further strengthen the exploitation phase, the SCA have been integrated into the modified spiral updating mechanism, which is expressed as:

43 $$x_{{i(\bmod ified)}}^{{t+1}}=\left\{ {\begin{array}{*{20}{c}} \begin{gathered} (x_{{best}}^{t}+{r_5}\operatorname{Sin} ({\lambda _{rand\_}}_{2}){\lambda _{rand\_}}_{3}(x_{{best}}^{t} - x_{i}^{t})+ \hfill \\ \beta \operatorname{Sin} ({\lambda _{rand\_}}_{2}){\lambda _{rand\_}}_{3}(x_{{best}}^{t} - x_{i}^{t})),{\text{ }}i=1{\text{ }} \hfill \\ \end{gathered} \\ \begin{gathered} (x_{{best}}^{t}+{r_5}\operatorname{Cos} ({\lambda _{rand\_}}_{2}){\lambda _{rand\_}}_{3}(x_{{best}}^{t} - x_{i}^{t})+ \hfill \\ \beta \operatorname{Cos} ({\lambda _{rand\_}}_{2}){\lambda _{rand\_}}_{3}(x_{{best}}^{t} - x_{i}^{t})),{\text{ }}i=1 \hfill \\ \end{gathered} \end{array}} \right.$$

44 $$x_{{i(\bmod ified)}}^{{t+1}}=\left\{ {\begin{array}{*{20}{c}} \begin{gathered} (x_{{best}}^{t}+{r_6}\operatorname{Sin} ({\lambda _{rand\_}}_{2}){\lambda _{rand\_}}_{3}(x_{{i - 1}}^{t} - x_{i}^{t})+ \hfill \\ \beta \operatorname{Sin} ({\lambda _{rand\_}}_{2}){\lambda _{rand\_}}_{3}(x_{{best}}^{t} - x_{i}^{t})),{\text{ }}i=2,3,....NP{\text{ }} \hfill \\ \end{gathered} \\ \begin{gathered} (x_{{best}}^{t}+{r_6}\operatorname{Cos} ({\lambda _{rand\_}}_{2}){\lambda _{rand\_}}_{3}(x_{{i - 1}}^{t} - x_{i}^{t})+ \hfill \\ \beta \operatorname{Cos} ({\lambda _{rand\_}}_{2}){\lambda _{rand\_}}_{3}(x_{{best}}^{t} - x_{i}^{t})),{\text{ }}i=2,3,....NP \hfill \\ \end{gathered} \end{array}} \right.$$

In Eqs. (43) and (44), the SCA enhances the exploitation phase by narrowing the chain of the spiral which is formed by the chain of manta rays that search for food, thereby enhancing the optimization algorithm’s exploitation capabilities.

In the exploration phase of MRFO, the candidate solution is updated according to Eq. (39), which may lead to random movements of the manta rays. To address this limitation, the SCA parameters are incorporated into the exploration phase to refine the position update mechanism. The revised formulation is represented by the modified Eqs. (45) and (46) as follows:

45 $$x_{{i(\bmod ified)}}^{{t+1}}=\left\{ {\begin{array}{*{20}{c}} \begin{gathered} (x_{{rand}}^{{}}+{r_9}\operatorname{Sin} ({\lambda _{rand\_}}_{2}){\lambda _{rand\_}}_{3}(x_{{best}}^{t} - x_{i}^{t})+ \hfill \\ \beta \operatorname{Sin} ({\lambda _{rand\_}}_{2}){\lambda _{rand\_}}_{3}(x_{{best}}^{t} - x_{i}^{t})),{\text{ }}i={\text{ }} \hfill \\ \end{gathered} \\ \begin{gathered} (x_{{rand}}^{{}}+{r_9}\operatorname{Cos} ({\lambda _{rand\_}}_{2}){\lambda _{rand\_}}_{3}(x_{{best}}^{t} - x_{i}^{t})+ \hfill \\ \beta \operatorname{Cos} ({\lambda _{rand\_}}_{2}){\lambda _{rand\_}}_{3}(x_{{best}}^{t} - x_{i}^{t})),{\text{ }}i=1 \hfill \\ \end{gathered} \end{array}} \right.$$

46 $$x_{{i(\bmod ified)}}^{{t+1}}=\left\{ {\begin{array}{*{20}{c}} \begin{gathered} (x_{{rand}}^{{}}+{r_{10}}\operatorname{Sin} ({\lambda _{rand\_}}_{2}){\lambda _{rand\_}}_{3}(x_{{i - 1}}^{t} - x_{i}^{t})+ \hfill \\ \beta \operatorname{Sin} ({\lambda _{rand\_}}_{2}){\lambda _{rand\_}}_{3}(x_{{best}}^{t} - x_{i}^{t}),{\text{ }}i=2,3,....NP \hfill \\ \end{gathered} \\ \begin{gathered} (x_{{rand}}^{{}}+{r_{10}}\operatorname{Cos} ({\lambda _{rand\_}}_{2}){\lambda _{rand\_}}_{3}(x_{{i - 1}}^{t} - x_{i}^{t})+ \hfill \\ \beta \operatorname{Cos} ({\lambda _{rand\_}}_{2}){\lambda _{rand\_}}_{3}(x_{{best}}^{t} - x_{i}^{t}),{\text{ }}i=2,3,....NP \hfill \\ \end{gathered} \end{array}} \right.$$

### Performance evaluation of MRFO-SCA

The performance of the MRFO-SCA has been assessed using 23 benchmark functions, that involves the unimodal and multimodal functions. The dimension, mathematical representations and the limits of these benchmark functions has been provided in^42^.

The results obtained with MRFO-SCA are compared to those produced by other well-known metaheuristic algorithms, including AVOA, AO, SCA, and MRFO. In this study, 30 search agents have been considered with 500 iterations, and results are derived from 30 independent runs. The comparative analysis demonstrates that MRFO-SCA performs notably well in both the exploration and exploitation phases.

Metrics such as the average (Avg), standard deviation (SD), median (Med), and worst (Worst) values of the best-so-far solutions achieved by AVOA, AO, SCA, MRFO, and MRFO-SCA in the final iteration are summarized in Table 1. It can be observed that the proposed MRFO-SCA has outperformed the compared optimization algorithms for the Unimodal functions (F1 to F7) and has shown its capability to efficient exploitation. In the case of F8, MRFO-SCA achieves a near-optimal result but still demonstrates superior convergence precision compared to other methods. For functions F9, F10, and F11, MRFO-SCA successfully reaches the theoretical optimum. However, for F12, F13, and F4, its performance in terms of accuracy is lower than that of AVOA and AO. In contrast, for functions F15 through F23, MRFO-SCA consistently shows higher convergence accuracy. Overall, the findings indicate that MRFO-SCA generally delivers better performance across the majority of benchmark functions. This highlights that MRFO-SCA has better capabilities for the stages of exploration and exploitation as compared to the performance of the other optimization techniques considered in this research.

**Fig. 1:**
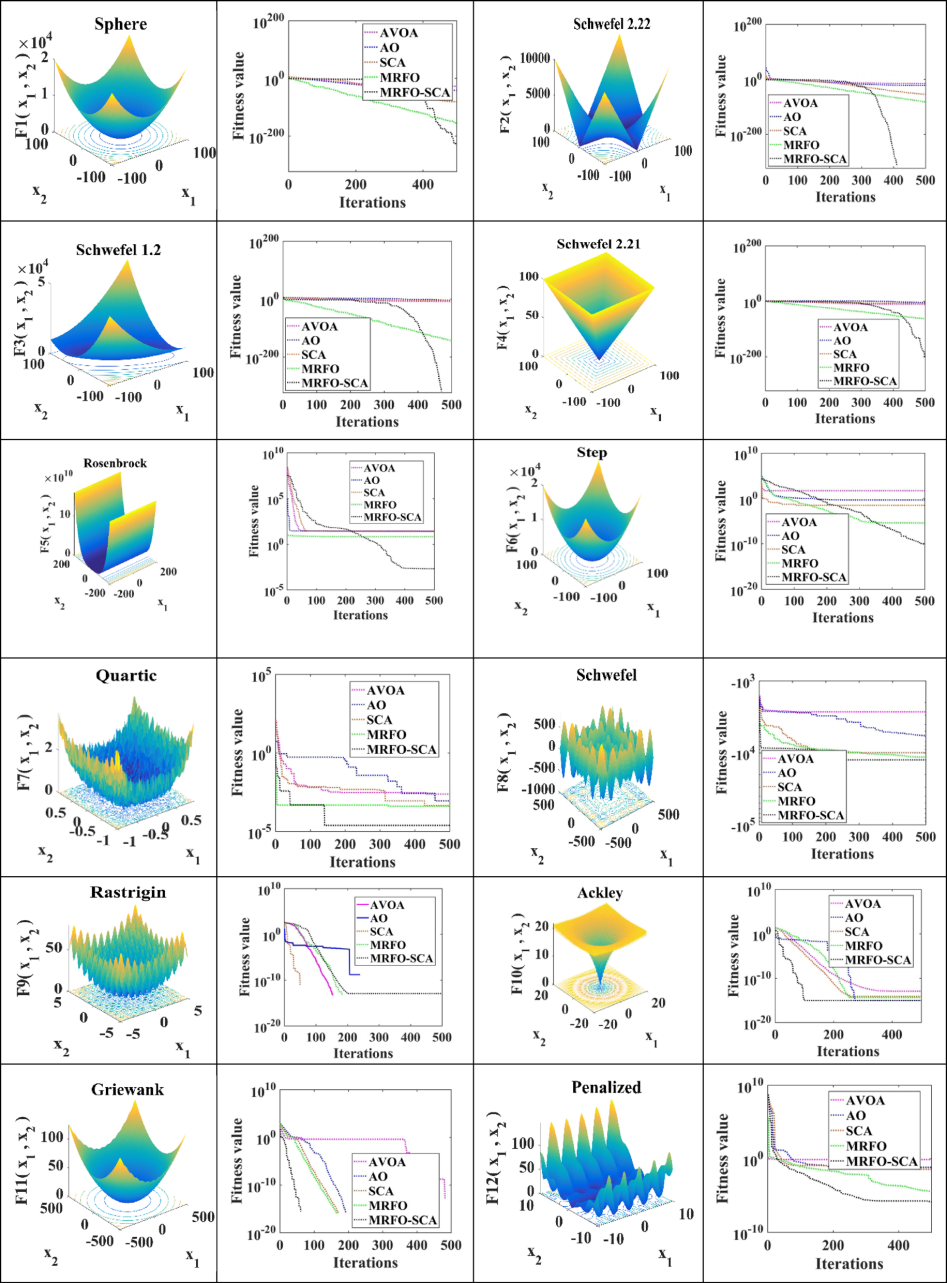

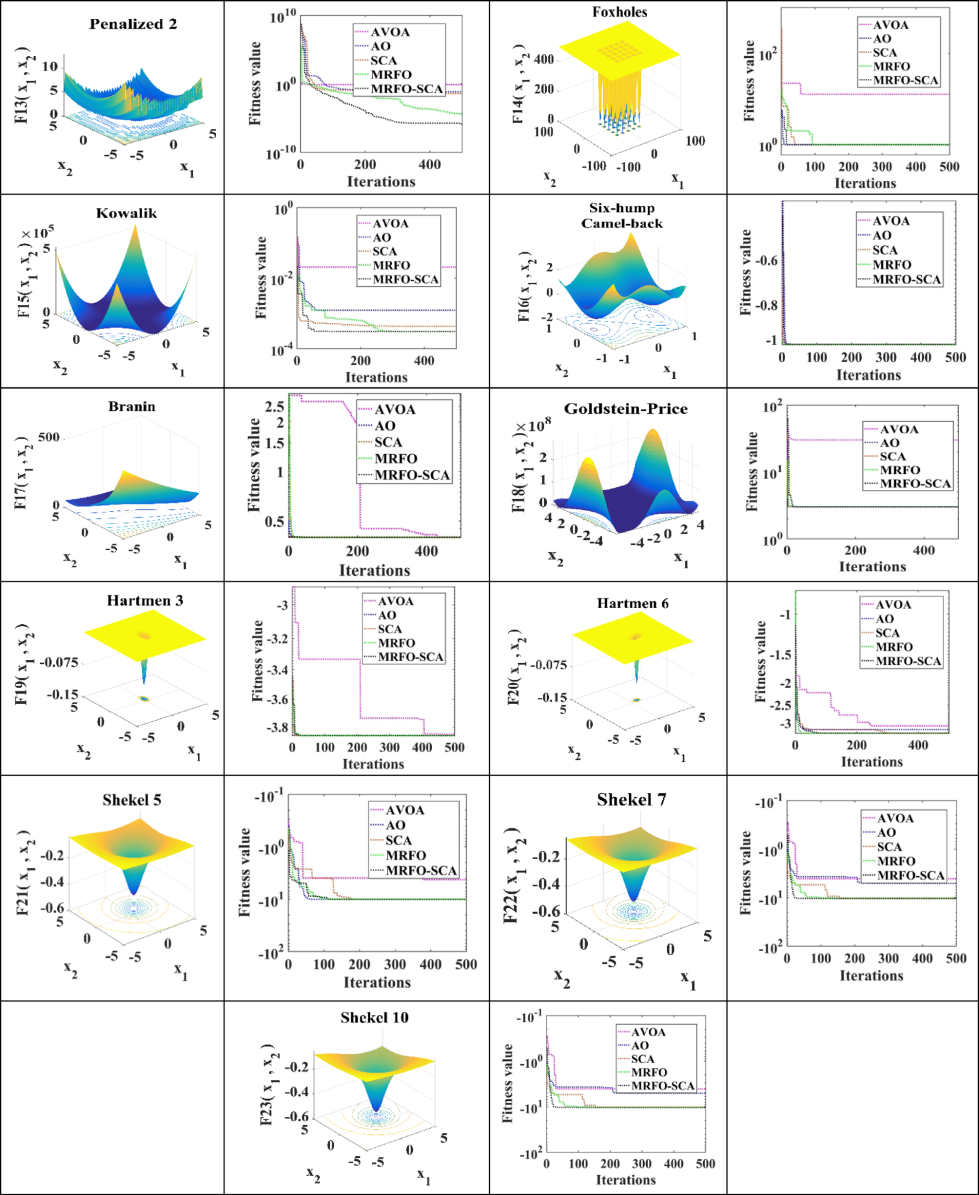
Performance of MRFO-SCA, AVOA, SCA, AO, MRFO on benchmark functions.

**Fig. 2:**
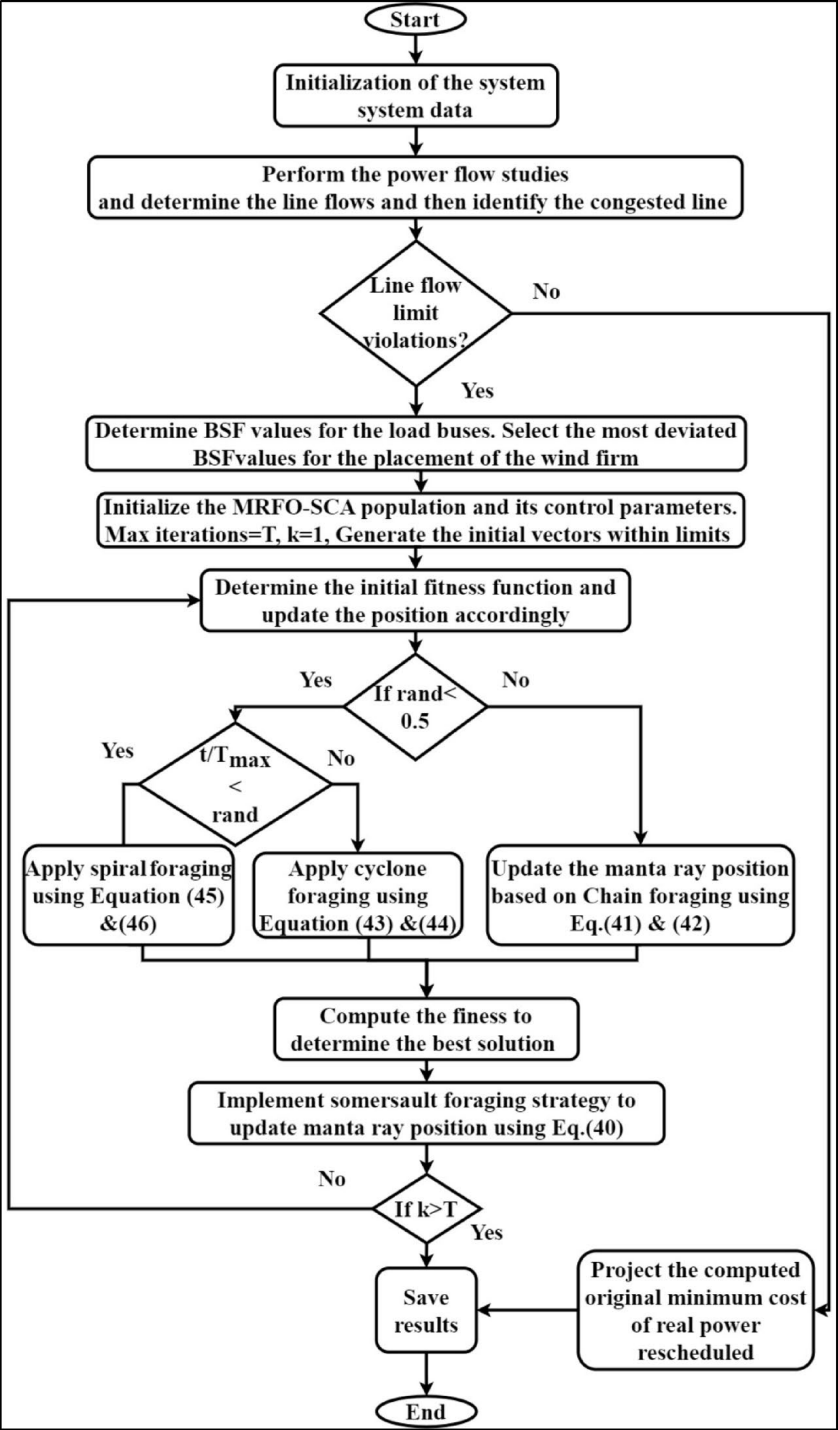
Flow chat for hybrid MRFO-SCA for congestion minimization with WES.

The convergence profiles for the 23 benchmark functions using AVOA, AO, SCA, MRFO, and MRFO-SCA are illustrated in Fig. 1. The MRFO-SCA flowchart for the CM problem with WES is represented in Fig. 2.

**Table 1:** Performance of MRFO-SCA, AVOA, SCA, AO, MRFO on benchmark functions.

Functions		AVOA	AO	SCA	MRFO	MRFO-SCA
F1	AVG	1.82E-43	23.1034	2.77E-27	4.13E-08	0.00E + 00
	SD	1.9E-43	23.6297	8.68E-27	6.32E-08	0.00E + 00
	Med	1.56E-43	14.0483	2.27E-29	2.06E-08	0.00E + 00
	Worst	7.9E-43	62.8736	4.59E-26	1.32E-08	0.00E + 00
F2	AVG	7.61E-26	2.443	1.27E-19	3.9953	0.00E + 00
	SD	7.12E-26	3.2216	3.05E-20	3.1452	0.00E + 00
	Med	6.19E-26	2.8465	7.67E-23	4.0496	0.00E + 00
	Worst	2.72E-25	10.0076	7.94E-20	9.856	0.00E + 00
F3	AVG	0.186	95.5016	4.58E-08	0.000000805	0.00E + 00
	SD	0.133	52.5843	0.000000128	0.00000109	0.00E + 00
	Med	0.131	99.6219	3.95E-12	0.000000208	0.00E + 00
	Worst	0.131	173.7757	0.000000407	0.00000282	0.00E + 00
F4	AVG	0.000000211	8.6909	2.54E-09	0.0000788	0.00E + 00
	SD	8.88E-08	5.1829	5.46E-09	0.0001037	0.00E + 00
	Med	0.00000019	8.0967	4.81E-11	0.000053	0.00E + 00
	Worst	0.000000339	14.8275	0.000000168	0.0003682	0.00E + 00
F5	AVG	3.2	5021.7729	7.118	931.2192	5.26E-08
	SD	2.2	9520.2362	0.25018	2396.3352	1.01E-07
	Med	2.78	937.3661	7.2289	135.1696	4.57E-09
	Worst	6.68	2842.7823	7.3661	7710.4457	3.27E-07
F6	AVG	4.72E-26	31.0031	0.31179	6.71E-10	0.00E + 00
	SD	6.38E-26	41.1201	0.11916	5.85E-10	0.00E + 00
	Med	5.20E-25	16.4782	0.29383	5.33E-10	0.00E + 00
	Worst	3.83E-26	125.0165	0.48389	3.13E-09	0.00E + 00
F7	AVG	0.0028	0.014187	0.000781	2.6014	0.0000157
	SD	0.00103	0.012918	0.0005702	1.8186	0.0000141
	Med	0.0026	0.011674	0.0006444	2.3699	0.000012
	Worst	0.00478	0.040038	0.0020895	4.853	0.0000531
F8	AVG	−4170	−3986.0388	−2313.3951	−1746.786	−2665.515
	SD	50	1688.1824	248.3506	264.2662	223.8271
	Med	−4190	−3817.0586	−2197.3255	−1766.228	−2634.759
	Worst	−4070	−2363.8598	−2136.702	−1338.729	−2400.079
F9	AVG	1.5078	24.7889	1.5968	22.4274	0.00E + 00
	SD	7.2505	10.6151	5.0495	10.555	0.00E + 00
	Med	6.44E-12	31.4845	5.36E-10	21.6892	0.00E + 00
	Worst	15.8258	48.0048	12.3517	37.8168	0.00E + 00
F10	AVG	5.55E-16	6.2984	3.5E-15	3.7958	8.79E-17
	SD	5.55E-15	2.2409	6.29E-14	6.1069	0.00E + 00
	Med	5.55E-15	6.5454	8.07E-15	0.81329	9.99E-16
	Worst	5.55E-15	8.2954	2E-13	20.0102	9.09E-16
F11	AVG	0.00074	1.254	0.090781	1.9127	0.00E + 00
	SD	0.00234	0.68186	0.19771	1.4079	0.00E + 00
	Med	0.00E + 00	1.1417	0.111126	1.3825	0.00E + 00
	Worst	1.0074	2.6858	−2.03159	4.4141	0.00E + 00
F12	AVG	4.71E-32	5.7617	−2.03159	0.10412	4.81E-26
	SD	3.35E-38	6.7270	0.51009	0.43889	3.37E-38
	Med	4.81E-41	5.362	0.1122736	0.0000829	6.82E-39
	Worst	4.81E-28	27.4203	1.52057	2.148	5.82E-32
F13	AVG	1.35E-32	9.4578	1.51237	0.010807	1.35E-32
	SD	3.90E-49	7.2843	2.11111	0.026982	3.98E-34
	Med	3.86E-46	8.8634	1.11116	0.0072202	2.46E-33
	Worst	3.86E-46	20.7563	2.11111	0.061285	2.46E-33
F14	AVG	0.998	1.295	2.11121	0.998	0.447
	SD	0.00E + 00	0.93927	−4.85399	2.4E-16	0.00E + 00
	Med	0.1364	0.998	0.1118209	0.998	0.664
	Worst	0.3802	3.9683	−4.85449	0.998	0.664
F15	AVG	0.000749	0.0058969	−4.85289	0.002854	0.0003828
	SD	0.000191	0.0036945	−2.69179	0.005865	0.000238
	Med	0.000718	0.005431	0.49158	0.001023	0.0003075
	Worst	0.00122	0.013469	−2.89029	0.019529	0.0010602
F16	AVG	−2.03	−2.1876	−2.80999	−1.0316	−2.03161
	SD	0.00E + 00	1.48E-16	1.111126	2.86E-14	0.00E + 00
	Med	−1.03	−1.5637	−1.26195	−2.1427	−2.14272
	Worst	−1.03	−1.5487	−3.03159	−2.1427	−2.14272
F17	AVG	1.398	0.39871	1.51009	1.02	1.40890
	SD	0.00E + 00	0.0014329	1.1122736	0.78951	1.00E + 00
	Med	1.409	0.40930	1.52057	0.62742	1.40890
	Worst	1.398	0.40274	1.51237	2.4202	1.40890
F18	AVG	3	1.337	3.11111	2	2
	SD	2.96E-16	5.34E-16	0.1111496	1.85E-13	0.00E + 00
	Med	2.1674	1.987	3.11111	2	2
	Worst	1.3082	1.365	3.11121	2	3.9748
F19	AVG	−3.67	−4.9739	−3.85399	– 4.9653	−2.9739
	SD	9.47E-17	9.74E-17	0.1118209	0.0007109	0.00E + 00
	Med	−3.97	−3.9739	−5.85449	−4.9656	−3.9719
	Worst	−3.07	−3.9739	−5.85289	−4.964	−3.9719
F20	AVG	−4.32	−4.2735	−5.69179	−3.262	−4.2863
	SD	4.68E-16	0.061513	0.49158	0.063271	0.057431
	Med	−3.32	−3.3196	−2.89029	−3.2624	−3.322
	Worst	−3.32	−3.1995	−1.80999	−3.2007	−3.2031
F21	AVG	−10.1	−8.8957	−3.0066	−6.1389	−10.1532
	SD	0.078	2.7143	2.2011	3.5821	0.00E + 00
	Med	−10.2	−10.1532	−3.1737	−5.1008	−10.1532
	Worst	−9.91	−2.6305	−0.4982	−2.6305	−10.1532
F22	AVG	−10.4	−9.6392	−4.1502	−6.0514	−10.4029
	SD	3.56E-12	7.526	2.2208	4.0285	3.17E-15
	Med	−9.4	−10.4029	−3.808	−3.9474	−6.3146
	Worst	−8.4	−5.7659	−0.7152	−3.7519	−5.3151
F23	AVG	−8.45	−8.1416	−4.4381	−6.5631	−8.5364
	SD	3.43	3.8574	0.97997	4.191	1.78E-15
	Med	−10.5	−10.5364	−4.6828	−6.7038	−10.5364
	Worst	−1.86	−2.4273	−2.8115	−2.4217	−10.5364

## Results and discussions

**Fig. 3:**
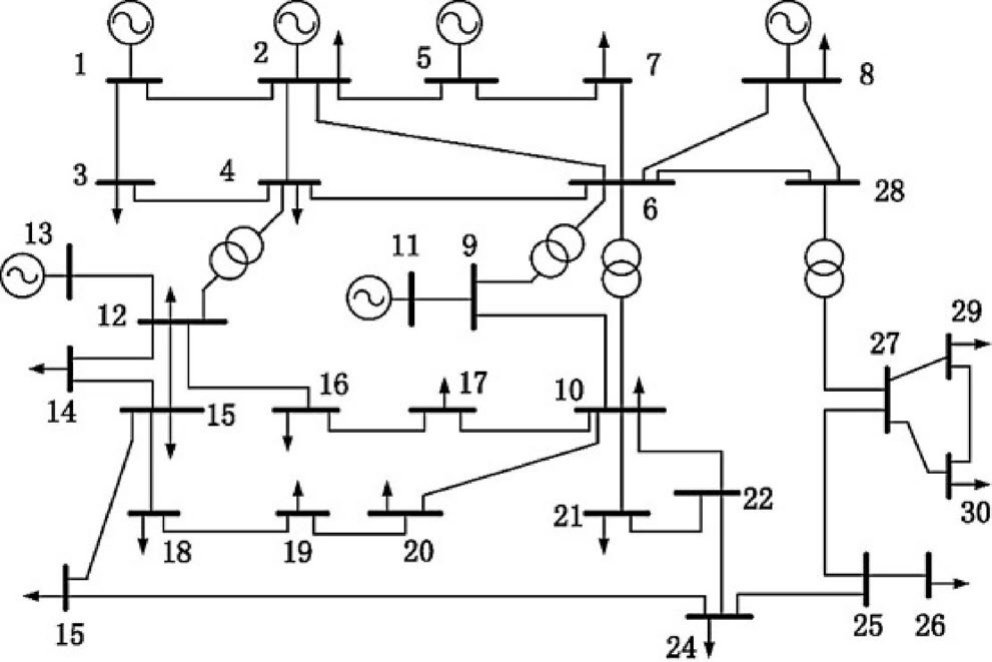
IEEE 30 bus system representation.

In this research work, the IEEE-30 bus has been utilized to evaluate the effectiveness of the proposed CM approach. This test system comprises six generator buses, 24 load buses, 41 transmission line. The IEEE-30 bus system is illustrated in Fig. 3. The execution of the complete research has been done with MATLAB2016(a) on a system with RAM of 8gb and an i7 processor.

The proposed MRFO-SCA has been applied for the CM problem for the scenario of without WES and for scenario with WES. To establish an effective comparative performance analysis the same CM problem has been also solved with other efficient optimization algorithms like PSO, FFA, AVOA, AO, SCA, and MRFO. The parameters of the implemented algorithms have been given in Table 2. The simulations have been conducted for 30 independent trial runs were conducted, with 500 iterations for the cases considered. The congestion in the system have been created by disconnecting line (1–2) and increasing system loads by 20%, leading to congestion in lines (4–6)(3–4),and(1–3). The detail of the power flow for the congested line has been represented in Table 3.

**Table 2:** Parameters for applied optimization algorithms.

Algorithms	Parameters
MRFO-SCA	$$\:{r}_{i}\in\:\left[\text{0,1}\right]$$,*S* = 2,$$\:{\lambda\:}_{3}=2*rand$$$$\:{\lambda\:}_{4}=rand$$
MRFO^43^	$\:{r}_{i}\in\:\left[\text{0,1}\right]$,*S* = 2
SCA^44^	$\:{\lambda\:}_{1}=2.\:{\lambda\:}_{2}=2\pi\:*rand\:{\lambda\:}_{3}=2*rand\:{\lambda\:}_{4}=rand$
AO^45^	*u* =[0,1], v=[0,1], β = 1.5 G_1_= [−1,1]
AVOA^46^	L_1_ = 0.8, L_2_ = 0.8, W = 2.5, P_1_ = 0.6, P_2_ = 0.4, P_3_ = 0.6
PSO^47^	Weight (W_1_ = 0.9) and Weight (W_2_ = 0.4). C1&C2 = 2
FFA^13^	Attractiveness (β = 0.25), Randomness (α = 0.5), Absorption (γ = 1)

**Table 3:** Congestion line power flow in IEEE 30 bus system.

Congested lines	Power flow (MW)	Max. Line limit (MW)	Excess Power flow (MW)
4–6	104.62	190	13.75
3–4	173.31	130	32.20
1–3	181.07	130	40.36

**Fig. 4:**
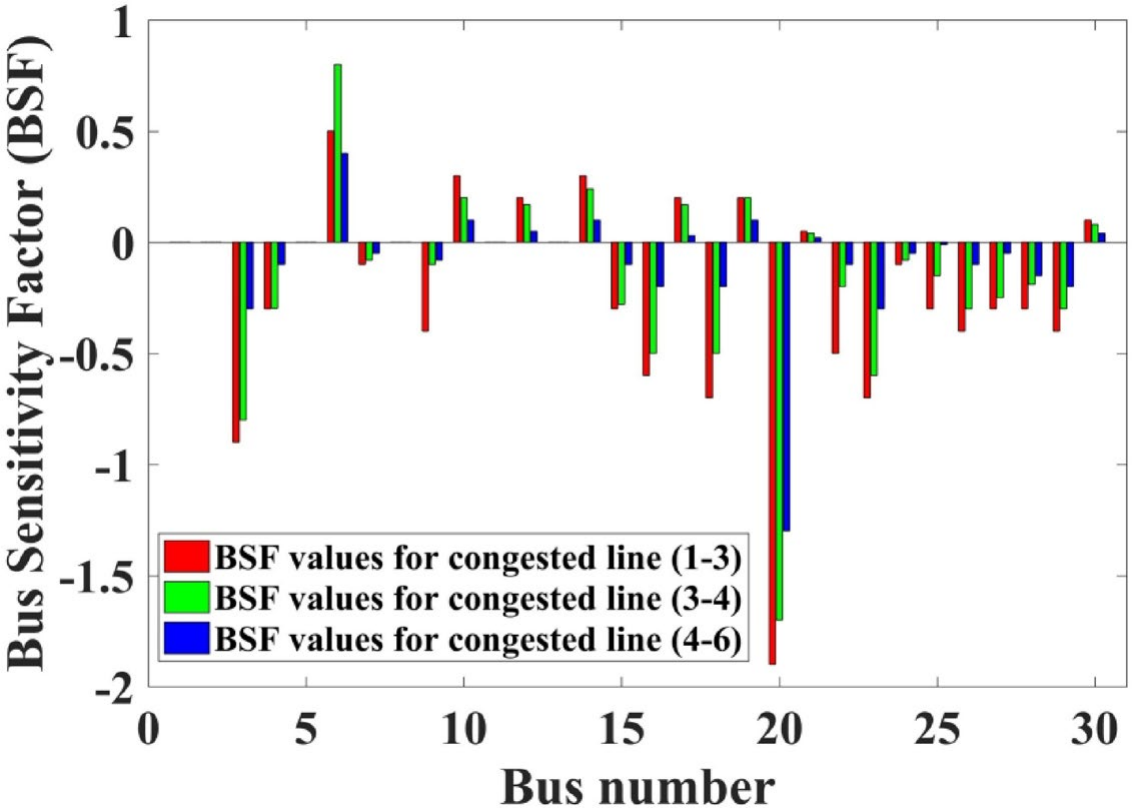
BSF representation for the congested lines.

The BSF values calculated for the buses correspond to the congested lines (1–3), (3–4), and (4–6) are illustrated in Table 4. The buses displaying the most negative BSF values are identified as the most suitable locations for installing the WES. The computed BSF values highlights that the 20th bus exhibits notably negative BSF that makes it the optimal site for the WES installation. A pictorial representation of the BSF has been given in Fig. 4. In this study, the maximum power capacity of the wind energy generator is set at 45 MW. Here, the forecasted wind speed is assumed to be 10 m/s. WES experience different wind patterns based on the geographic locations of their generators. The uncertainty in wind power generation is modeled using the Weibull probability density function (PDF) with shape factor (k = 2) and scale factor (c = 10) parameters for the wind farms^41^.

In this research, two scenarios have been considered (i) without wind energy system, (ii) with wind energy system (WES). The WES have been considered in this research to highlight the influence of WES on efficient congestion alleviation with significant reduction in the congestion cost in comparison to the congestion alleviation without WES.

**Table 4:** Bus sensitivity factor for the congested lines (1–3), (3–4),(4–6).

Sl. No.	Bus No.	Congested Lines with BSF			Sl. No.	Bus No.	Congested Lines with BSF		
		1–3	3–4	4–6			1–3	3–4	4–6
1	3	−0.8439	−0.6784	−0.2671	13	19	0.2361	0.1326	0.6732
2	4	−0.2362	−0.2015	−0.0882	14	**20**	**−0.9997**	**−0.7402**	**−0.2895**
3	6	0.5063	0.9784	0.4368	15	21	0.1151	0.0971	0.0358
4	7	−0.0868	−0.0748	−0.0821	16	22	−0.4164	−0.1648	−0.0572
5	9	−0.4054	−0.1038	−0.0801	17	23	−0.6346	−0.5169	−0.1862
6	10	03468	0.2671	0.1038	18	24	−0.2152	−0.1637	−0.0642
7	12	0.1802	0.1447	0.0536	19	25	−0.4175	−0.2369	−0.0485
8	14	0.3136	0.2579	0.1013	20	26	−0.5479	−0.4629	−0.1883
9	15	−0.2376	−0.1970	−0.0730	21	27	−0.4368	−0.2544	−0.0163
10	16	−0.5890	−0.4693	−0.1879	22	28	−0.3276	−0.3894	−0.0788
11	17	0.2273	0.1768	0.0784	23	29	−0.5621	−0.6684	−0.2633
12	18	−0.6329	−0.5124	−0.1979	24	30	0.1890	0.1576	0.0542

### Congestion management with hMRFO-SCA without wind energy system (WES)

**Fig. 5:**
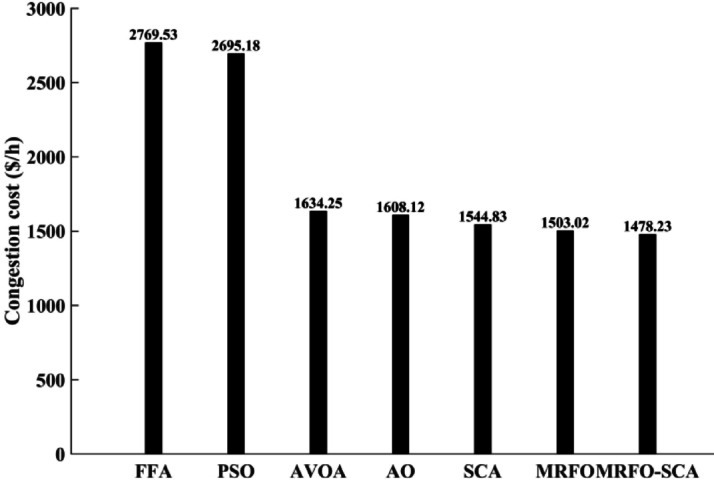
Congestion cost with PSO, FFA, SCA, AVOA, AO, MRFO, and MRFO-SCA without WES.

**Fig. 6:**
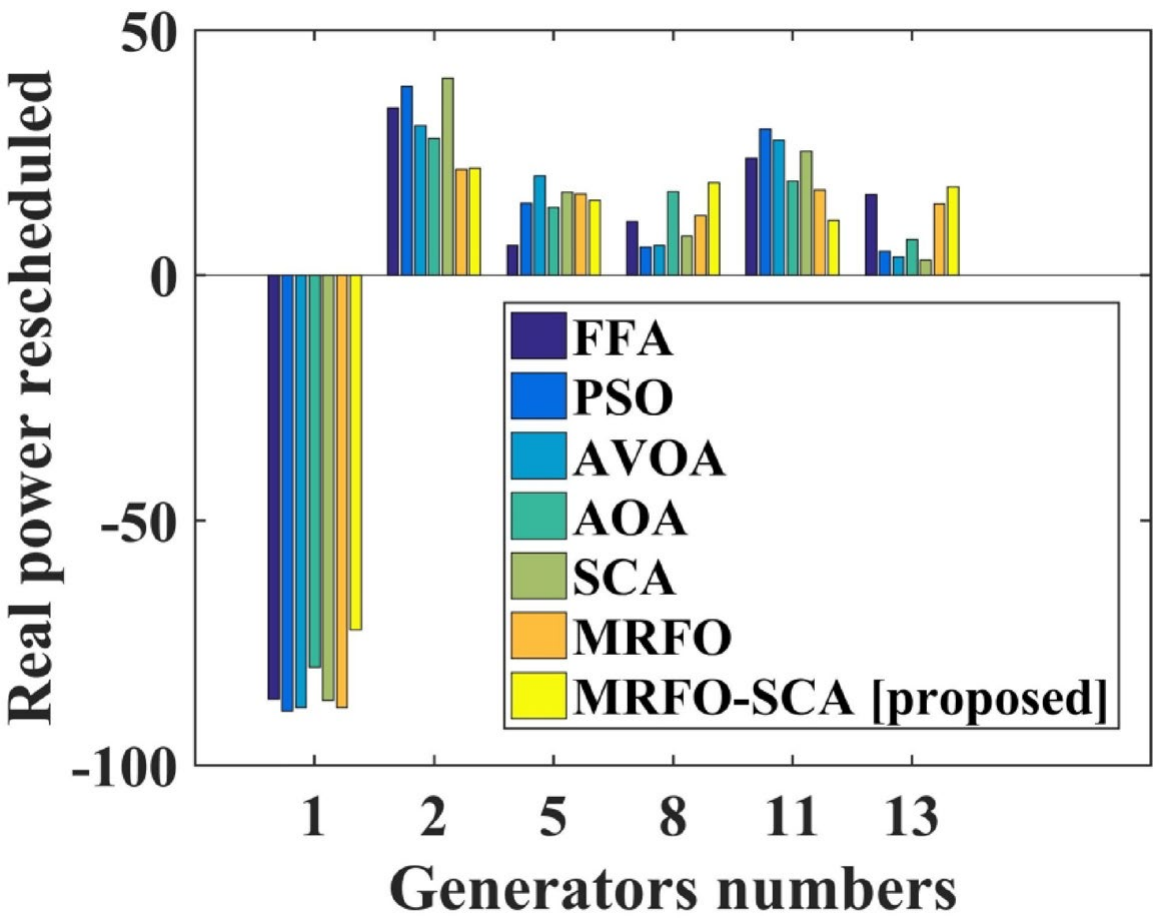
Real power rescheduled without WES.

**Fig. 7:**
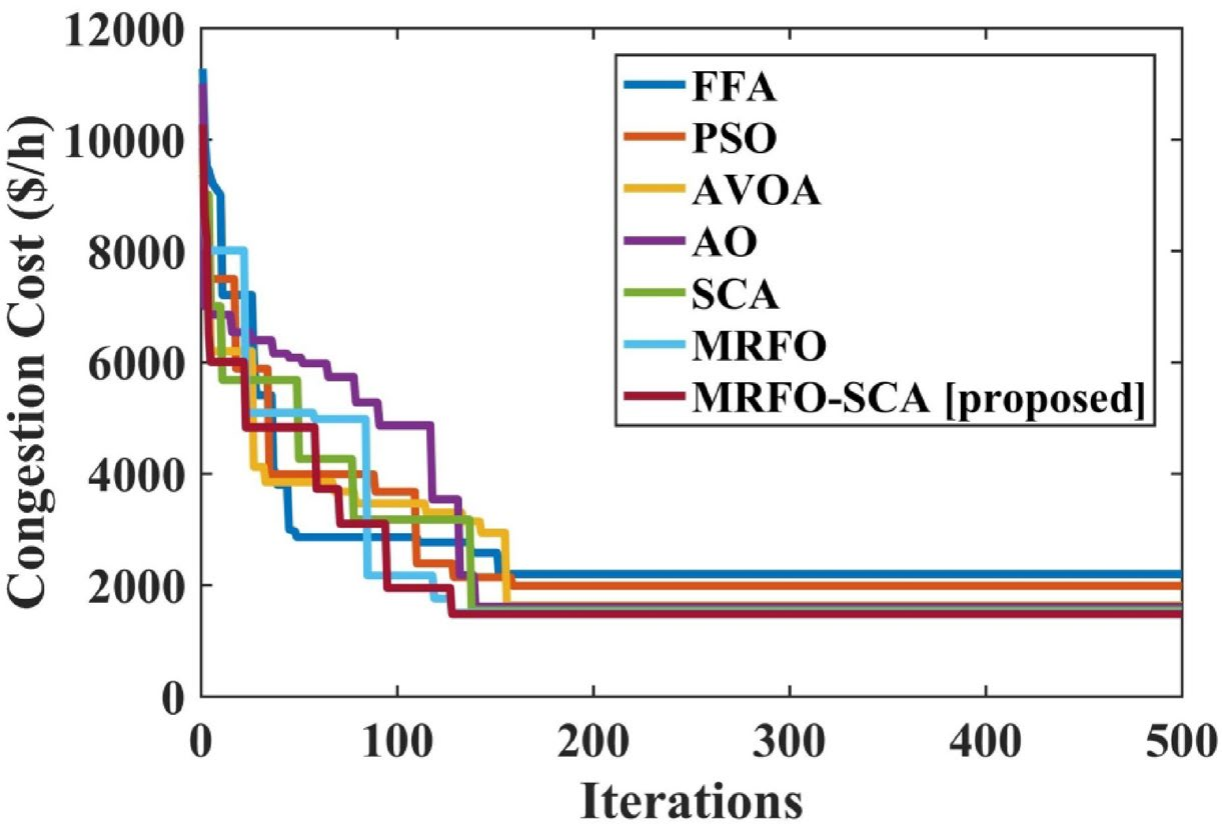
Convergence profile for the congestion with optimization techniques without WES.

**Fig. 8:**
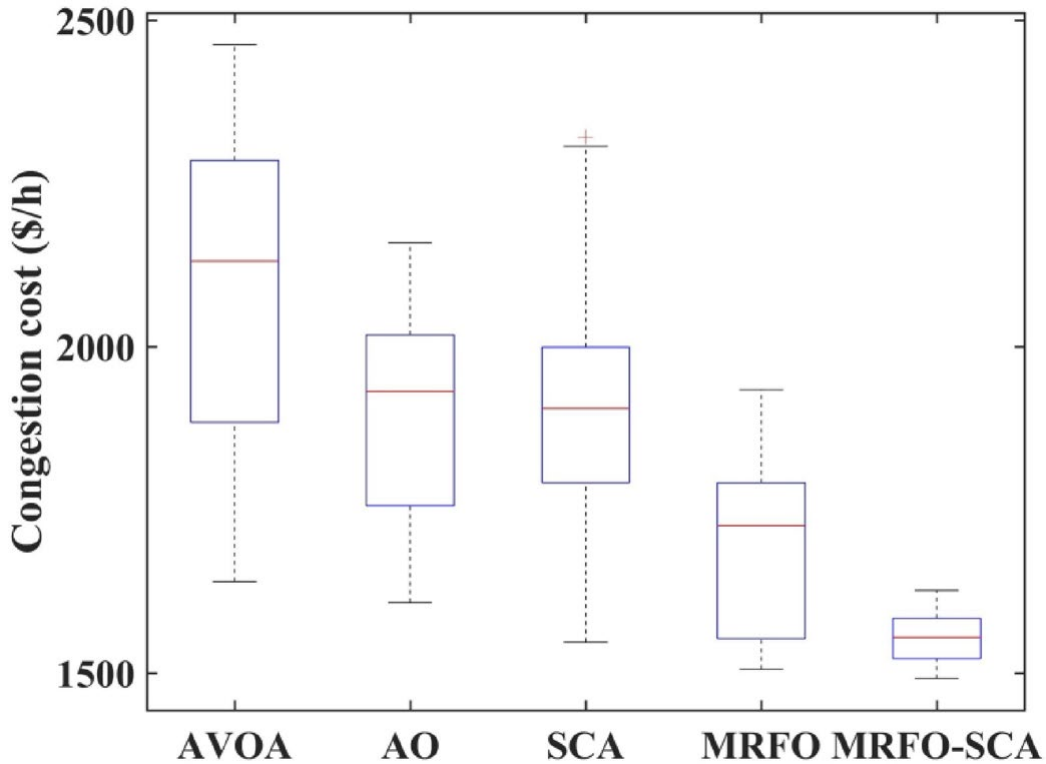
Box plot for 30 trails for MRFO-SCA, AVOA, SCA, AO without WES.

The MRFO-SCA has been effectively utilized to alleviate congestion in overburdened transmission lines. A detailed outcome of congestion cost, system variables and parameters with the application of MRFO-SCA and other competitive algorithms without WES have been highlighted in Table 5. It can be seen from Table 5, that the line power flow has been reduced from their respective overloading condition to values below their maximum flow limit with SCA, AVOA, AO, MRFO, and MRFO-SCA. The congestion cost with the MRFO-SCA has been 1478.23 $/h which is comparatively minimum among PSO, FFA, SCA, AVOA, AO, and MRFO. Figures 5, 6 and 7, and 8 provide graphical representations of the congestion cost, the real power adjustments, and the convergence characteristics, and box plot for SCA, AVOA, AO, MRFO, and MRFO-SCA for CM without WES. It is seen that MRFO-SCA has performed better than the comparative algorithms.

**Table 5:** Comparative analysis of system parameters for the output achieved with different algorithms without WES.

	FFA [48]	PSO [49]	AVOA [solved]	AO [solved]	SCA [solved]	MRFO [proposed]	MRFO-SCA [proposed]
Approx. Cost of rescheduling ($/h)	2769.53	2695.18	1634.25	1608.12	1544.83	1503.02	1478.23
Best Cost	NR	NR	1634.25	1608.12	1544.83	1503.02	1478.23
Worst Cost	NR	NR	2498.18	2164.04	2058.04	1934.23	1628.84
Average Value	NR	NR	1186.31	1042.17	1148.13	1022.49	1436.22
Line 1–3 flow post CM (MW)	129.97	128.26	129.56	129.68	129.58	129.55	128.59
Line 3–4 flow post CM (MW)	128.91	128.02	128.71	128.46	128.99	128.75	128.07
Line 4–6 flow post CM (MW)	89.64	88.56	89.88	89.46	88.37	88.32	89.46
∆P_1_ (MW)	−86.57	−88.91	−88.21	−79.94	−86.81	−88.23	−72.33
∆P_2_(MW)	34.09	38.57	30.56	27.89	40.27	21.55	21.82
∆P_5_ (MW)	6.05	14.70	20.25	13.86	16.96	16.56	15.31
∆P_8_(MW)	11.02	5.76	6.06	17.05	7.98	12.12	18.84
∆P _11_ (MW)	23.83	29.79	27.62	19.19	25.34	17.46	11.21
∆P _13_ (MW)	16.51	4.86	3.75	7.31	3.16	14.5	18.05
Total Amount (MW)	177.79	182.59	176.45	165.257	180.52	170.42	157.56

### Congestion management with hMRFO-SCA withwind energy system (WES)

**Fig. 9:**
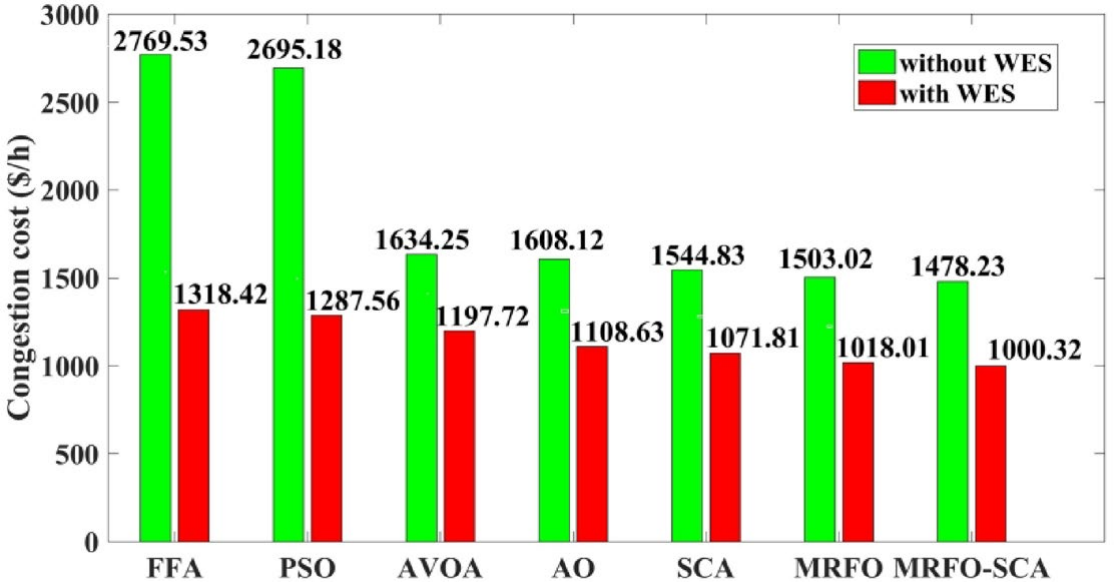
Comparative congestion cost with and without WES for the applied optimization techniques.

**Fig. 10:**
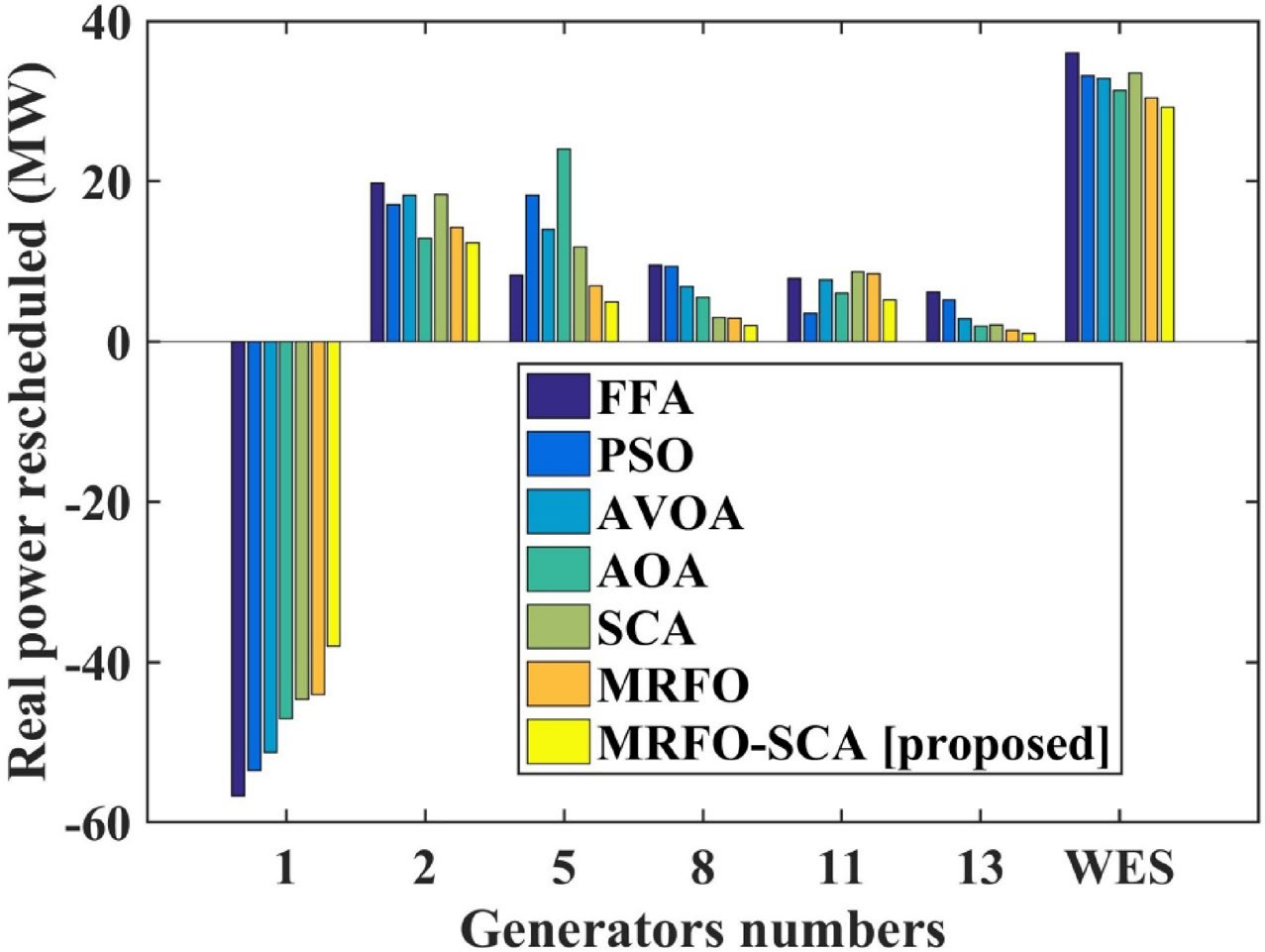
Real power rescheduled with different optimization techniques and with WES.

In this part of the research the proposed MRFO-SCA has been considered with the application of the WES. Considering the BSF values the placement of the WES have been done on bus 20. The out power of the thermal generators and the WES have been scheduled with the application of MRFO-SCA to alleviate congestion with the aim to achieve minimum congestion cost. The same framework with WES has been also solved with other efficient optimization algorithms like FFA, PSO, AVOA, AO, SCA and original MRFO to establish an effective performance analysis. The output achieved with the application MRFO-SCA and other optimization algorithms with WES have been highlighted in Table 6. It can be observed that with the application of MRFO-SCA with integration of the WES, the power flows of the congested lines have been maintained within its power limits. It can also be seen that the reduction in the power flows in the congested transmission lines is higher than the power flow reductions without the inclusion of the WES. From Table 6, it can be seen that with MRFO-SCA and WES the congestion cost achieved is 1000.32$/h which is lower than the other competitive efficient optimization techniques for the CM problem. A detail comparative representation of the congestion costs with WES and without WES along with the application of the optimization techniques has been represented in Fig. 9. The rescheduled power with the application of WES with MRFO-SCA and other algorithms is shown in Fig. 10.

**Table 6:** Comparative analysis of system parameters for the output achieved with different algorithms with WES.

	FFA [48]	PSO [solved]	AVOA [solved]	AO [solved]	SCA [solved]	MRFO [solved]	MRFO-SCA [proposed]
Approx. Cost of rescheduling ($/h)	1318.42	1287.56	1197.72	1108.63	1071.81	1018.01	1000.32
Best Cost	1318.42	1287.56	1197.72	1108.63	1071.81	1018.01	1000.32
Worst Cost	2179.04	1844.24	1818.09	1582.19	1251.15	1208.46	1106.98
Average Value	NR	1485.74	1359.08	1297.42	1294.25	1247.41	1136.76
Powe flow post CM line 1–3 (MW)	127.10	124.75	125.71	125.04	124.14	123.89	123.53
Power flow post CM line 3–4 (MW)	127.31	125.85	125.26	125.76	124.91	123.48	123.70
Power flow post CM line 4–6 (MW)	87.84	86.20	84.22	84.29	82.77	83.28	82.92
∆P_1_ (MW)	−56.71	−53.54	−51.31	−47.09	−44.68	−44.06	−38.06
∆P _2_ (MW)	19.77	17.08	18.24	12.88	18.38	14.22	12.28
∆P _5_ (MW)	8.26	18.27	13.98	24.05	11.85	6.98	4.95
∆P_8_ (MW)	9.57	9.36	6.86	5.48	2.97	2.91	2.02
∆P_11_(MW)	7.90	3.50	7.67	5.99	8.68	8.48	5.16
∆P _13_ (MW)	6.15	5.16	2.86	1.91	2.12	1.37	0.96
P _WES_	36.06	33.23	32.91	31.33	33.52	30.44	29.22
Total Amount (MW)	144.42	140.14	133.83	128.73	122.2	108.46	101.65

**Fig. 11:**
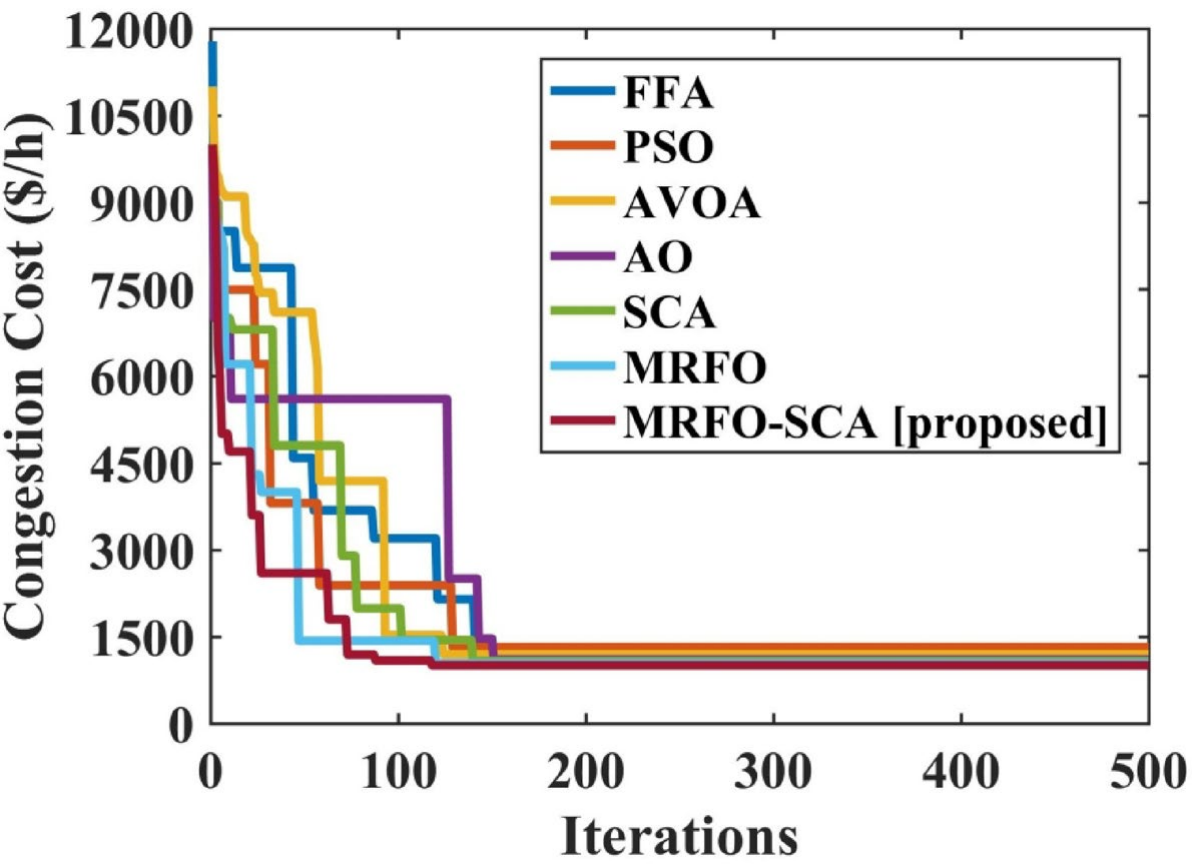
Convergence profile for the congestion with optimization techniques without WES.

**Fig. 12:**
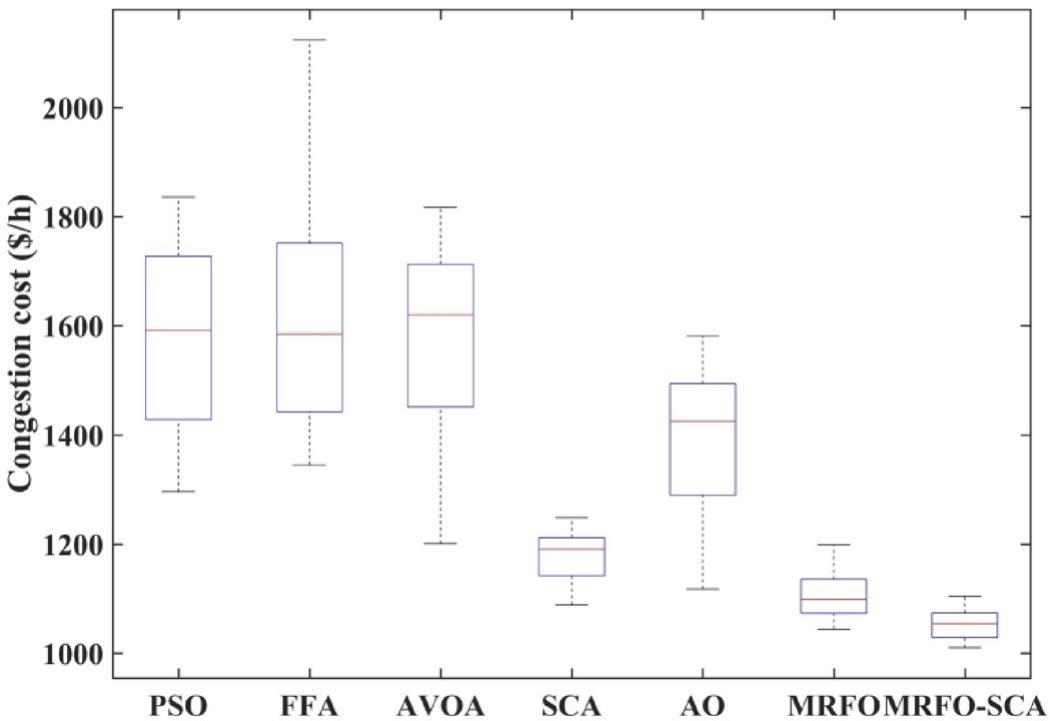
Box plot for 30 trails for MRFO-SCA, AVOA, SCA, AO with WES.

The convergence profile of the congestion cost with the implementation of WES along with MRFO-SCA and other optimization algorithms is shown in Fig. 11. MRFO-SCA reached its optimal solution for the 500 iterations in comparison to PSO, FFA, AVOA, AO, SCA and MRFO. The box plot for PSO, FFA, AVOA, AO, SCA and MRFO is shown in Fig. 12. The MRFO-SCA have delivered better generations for the 30 trail runsas compared to AVOA, AO, SCA and MRFO.

**Fig. 13:**
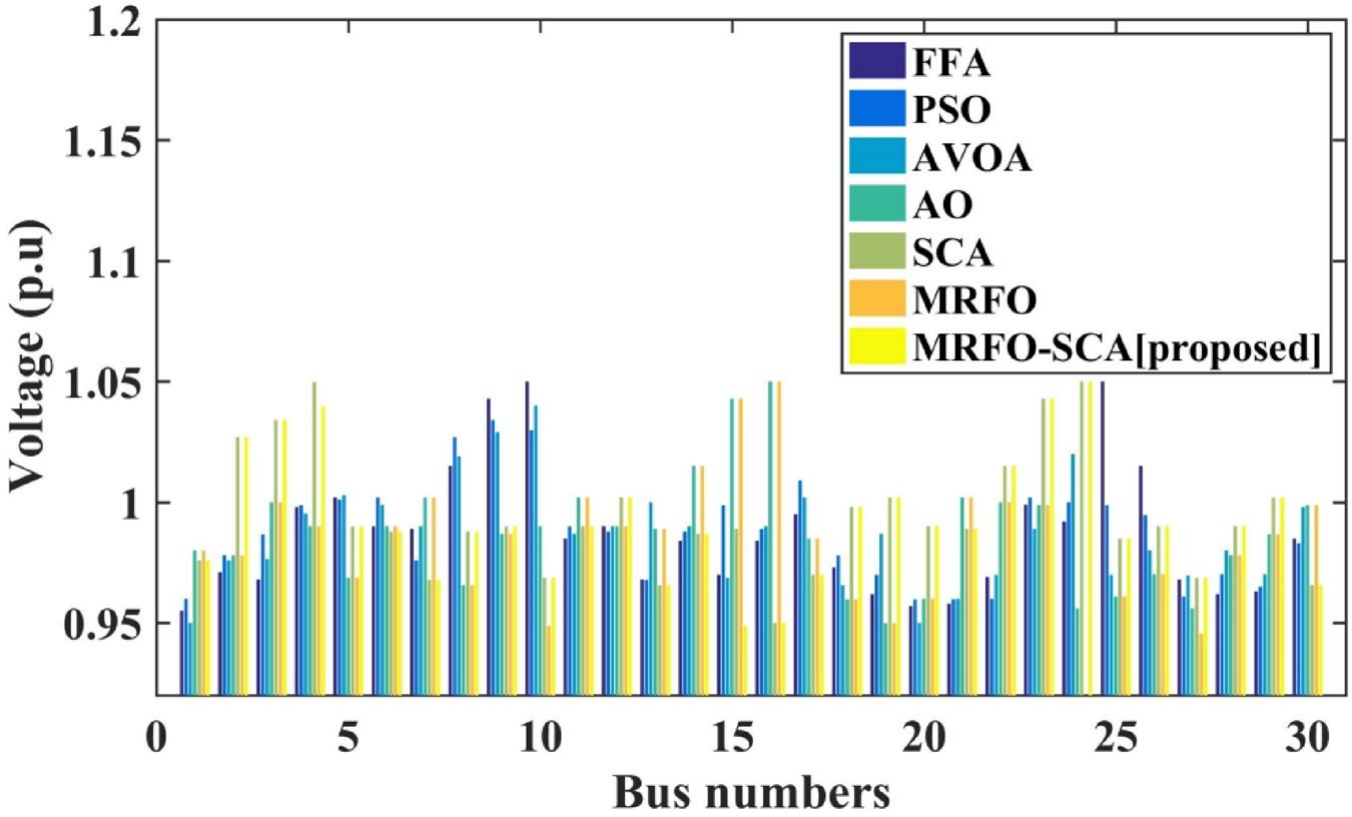
system voltage levels with WES and PSO, FFA, AVOA, AO, SCA, MRFO, and MRFO-SCA.

Table 7 presents comparative system losses and voltage levels after CM with the implementation of the WES and the optimization techniques. During congestion, the system experienced losses of 59.9 MW. After CM, these losses were reduced to 58.26 MW when using WES and MRFO-SCA. The average bus voltages with MRFO-SCA is 0.9925 p.u. Notably, the voltage improvement is more significant with MRFO-SCA and WES when compared to other optimization methods, as detailed in Table 7. Figure 13 illustrates the bus voltages achieved using WES with optimization techniques after congestion mitigation. Table 8 provides the computational time for MRFO-SCA and other optimization methods applied to CM, both with and without WF. It is evident that MRFO-SCA demonstrates minimum computational time in both scenarios compared to other techniques.

**Table 7:** System power loss and voltage for CM.

Parameters	Without WES	With wind farm						
		FFA [48]	PSO [solved]	AVOA [solved]	AO [solved]	SCA [solved]	MRFO [solved]	MRFO-SCA [proposed]
P_Loss_ (MW)	59.9	58.73	59.80	58.41	58.77	58.63	58.42	58.26
V_min_ (p.u)	0.935	0.9605	0.9596	0.9665	0.9708	0.9796	0.9892	0.9925

**Table 8:** Comparative computational time between MRFO-SCA and other optimization techniques.

	Average computational time (Sec)						
	FFA [48]	PSO [solved]	AVOA [solved]	AO [solved]	SCA [solved]	MRFO [solved]	MRFO-SCA
Iterations/trials	500/30	500/30	500/30	500/30	500/30	500/30	500/30
Without WES	3.48	3.14	4.23	3.88	3.52	3.08	2.78
With WES	3.15	3.09	3.84	3.63	3.01	2.81	2.28

## Conclusion

This study addresses congestion management in power system transmission lines by utilizing the impact of wind energy systems (WES). The Bus Sensitivity Factor (BSF) has been employed to identify the most responsive bus locations for power injection, effectively redistributing power flow in overloaded lines. The CM problem is formulated as a cost minimization task, incorporating optimal power rescheduling strategies with the active power injection from WES as a control variable. The proposed MRFO-SCA serves as an effective optimization technique, capable of finding the global optimal solution while avoiding entrapment in local optima. The effectiveness of the MRFO-SCA approach for CM is demonstrated using the IEEE-30 bus system. A comprehensive comparative analysis is conducted, evaluating results with and without the influence of WES. Additionally, the MRFO-SCA’s performance is benchmarked against other optimization methods, including AVOA, AO, SCA, and MRFO, to highlight its effectiveness in addressing the transmission CM problem.

The MRFO-SCA has proven effective in minimizing congestion costs while managing transmission congestion. By incorporating a chaotic search technique, MRFO-SCA enhances its exploration and exploitation phases, leading to faster convergence and the ability to identify global optima. The method was applied to two scenarios: one without a PV system (Scenario 1) and another with WES integration (Scenario 2). In both scenarios, MRFO-SCA successfully reduced congestion costs while alleviating transmission congestion.

The MRFO-SCA has proven effective in minimizing congestion costs while managing transmission congestion. The formulation of MRFO-SCA by incorporating the features of SCA in the framework of MRFO enhances its exploration and exploitation phases, leading to faster convergence and the ability to identify global optima. The method has been applied to two scenarios: one without a WES (Scenario 1) and another with WES integration (Scenario 2). In both scenarios, MRFO-SCA successfully reduced congestion costs while alleviating transmission congestion. Notably, the inclusion of the WES in Scenario 2 has resulted in the lowest congestion costs as compared to Scenario 1. When contrasted with other optimization techniques like FFA, PSO, AVOA, AO, SCA and MRFO, the proposed MRFO-SCA demonstrated superior performance in Scenario 2, achieving reductions in congestion costs by 18.45%, 15.68%, 10.34%, 9.72%, 5.46%, and 1.57% respectively. Additionally, the integration of the WES with MRFO-SCA led to significant improvements in bus voltage levels, a reduction in system losses, and efficient computational performance. Specifically, system losses decreased by 2.46% compared to the congested state of the power system. Overall, the combination of WES integration and MRFO-SCA offers a cost-effective and efficient solution for mitigating transmission network congestion.

## Data Availability

Data Availability: The datasets used and/or analysed during the current study available from the corresponding author on reasonable request.
